# Comparison between articular chondrocytes and mesenchymal stromal cells for the production of articular cartilage implants

**DOI:** 10.3389/fbioe.2023.1116513

**Published:** 2023-02-21

**Authors:** Nadine Frerker, Tommy A. Karlsen, Maria Stensland, Tuula A. Nyman, Simon Rayner, Jan E. Brinchmann

**Affiliations:** ^1^ Department of Immunology, Oslo University Hospital, Oslo, Norway; ^2^ Institute of Clinical Medicine, Faculty of Medicine, University of Oslo, Oslo, Norway; ^3^ Department of Medical Genetics, Oslo University Hospital, Oslo, Norway; ^4^ Hybrid Technology Hub—Centre of Excellence, Faculty of Medicine, University of Oslo, Oslo, Norway; ^5^ Department of Molecular Medicine, Faculty of Medicine, University of Oslo, Oslo, Norway

**Keywords:** articular cartilage, tissue engineering, chondrocytes, mesenchymal stromal (or stem) cells, microRNA

## Abstract

Focal lesions of articular cartilage give rise to pain and reduced joint function and may, if left untreated, lead to osteoarthritis. Implantation of *in vitro* generated, scaffold-free autologous cartilage discs may represent the best treatment option. Here we compare articular chondrocytes (ACs) and bone marrow-derived mesenchymal stromal cells (MSCs) for their ability to make scaffold-free cartilage discs. Articular chondrocytes produced more extracellular matrix per seeded cell than mesenchymal stromal cells. Quantitative proteomics analysis showed that articular chondrocyte discs contained more articular cartilage proteins, while mesenchymal stromal cell discs had more proteins associated with cartilage hypertrophy and bone formation. Sequencing analysis revealed more microRNAs associated with normal cartilage in articular chondrocyte discs, and large-scale target predictions, performed for the first time for *in vitro* chondrogenesis, suggested that differential expression of microRNAs in the two disc types were important mechanisms behind differential synthesis of proteins. We conclude that articular chondrocytes should be preferred over mesenchymal stromal cells for tissue engineering of articular cartilage.

## 1 Introduction

Focal lesions of articular cartilage are a common problem (Welton et al., 2018). Due to the limited intrinsic healing capacity of cartilage, even small defects may develop into osteoarthritis (OA) if left untreated. Different treatment strategies have been tried including mosaicplasty, microfracture, osteochondral autograft, and autologous chondrocyte implantation. However, the repair tissue frequently contains fibrocartilage instead of hyaline cartilage (Richter et al., 2015; Armiento et al., 2019). While pain is often reduced and functionality improved, the lesions tend to recur, leading eventually to the development of OA.

To overcome these problems new treatment options are being investigated based on the production of implantable tissue engineered cartilage. The disc shape normally chosen to simulate the shape of articular cartilage is frequently provided by natural or synthetic biomaterials, but the extracellular matrix (ECM) needs to be produced by cells in order to be as similar as possible to native hyaline ECM (Zhao et al., 2021a). The two most commonly used sources of cells are articular chondrocytes (ACs) and mesenchymal stromal cells (MSCs). Both cell types can be obtained from the patient, providing an autologous cell source. The procedure for obtaining ACs involves an arthroscopy directed biopsy of a piece of healthy articular cartilage, leaving an iatrogenic lesion with a small, but well described morbidity (Matricali et al., 2010). The procedure for obtaining MSCs normally involves a bone marrow (BM) aspiration from the posterior superior iliac crest, causing limited discomfort and no residual damage. Both cell types are easily expanded *in vitro*, involving approximately the same effort. Interestingly, both ACs and MSCs change in the course of *in vitro* culture (Shahdadfar et al., 2005; Karlsen et al., 2010), yielding cells with similar gene expression (Karlsen et al., 2019). Both cell types can be differentiated to ECM-producing chondrocytes using the same chondrogenic differentiation cocktail.

Experiments to identify which of the two cell types is preferable have been performed both *in vitro* and *in vivo*. Previously, using an alginate scaffold to make cartilage discs and characterising gene expression using an mRNA microarray assay we found similar properties between the cell types (Fernandes et al., 2013). A comparison of the chondrogenic potency of ACs with MSCs derived from BM, infrapatellar fat pad or subcutaneous fat, based on a small number of mRNA and cell surface and ECM synthesis markers, argued for ACs, followed by fat pad MSCs (Garcia et al., 2016). Conversely, animal studies of cartilage repair have tended to put MSCs ahead of ACs (Yan and Yu, 2007; Marquass et al., 2011). No human clinical trials directly comparing the two cell types have yet been published. Thus, this issue must be considered unresolved at this time.

Analysis of articular cartilage volume reveals that cells constitute <3% while the remainder consists of ECM molecules and water (Hunziker et al., 2002). The functionality of tissue-engineered cartilage, therefore, depends largely on the composition of the ECM. The regulation of ECM synthesis within the chondrocytes is complex and involves signalling pathways, transcription factors, and epigenetic changes among other factors (Kim et al., 2011; Duan et al., 2015; Neefjes et al., 2020). Recent advances in proteomics have enabled the quantification of several thousand proteins from biological samples, and this technique has been used in the analysis both of native healthy cartilage, OA cartilage, and engineered cartilage (Wu et al., 2007; Pu and Oxford, 2015; Hsueh et al., 2016). MicroRNAs (miRNAs) are a relatively recent addition to the list of factors regulating ECM synthesis. MiRNAs are short (_~_20–25 nt) non-coding RNA sequences that generally exert their effect by binding to the seed sequence (nt 2–8) of the 3′ UTR of mRNAs. The most common result is reduced protein levels, either through degradation of the mRNA or by interference with translation (Gebert and MacRae, 2019). However, protein levels may also be increased, most likely due to miRNA-mediated downregulation of inhibitors of the protein expression. MiRNAs are derived from precursor stem-loop structures which are trimmed in the nucleus and later in the cytosol to yield RNA duplexes consisting of a 5p and a 3p strand, named according to the side of the stem-loop from which they derive. Both strands exist as canonical versions, which are the sequences most commonly published for each miRNA, or isomiRs, which are sequence variants of the canonical miRNAs. IsomiRs which differ from the canonical miRNA in the seed sequence are most likely to show different functionality from the canonical miRNA (Al-Modawi et al., 2021). The role of individual canonical miRNAs during *in vitro* chondrogenesis has been described (Karlsen et al., 2016). However, a comprehensive analysis of the relationship between miRNA expression and the composition of engineered cartilage has, to the best of our knowledge, not previously been published.

In the current study we have directly compared ACs and BM-MSCs (referred to as MSCs in the following text) as cell candidates for tissue engineering of hyaline cartilage using a scaffold-free strategy to make cartilage discs (Frerker et al., 2021). We observed that ACs made thicker cartilage than MSCs, and propose an explanation for why this occurs. We also show by proteomics analysis that the ECM made by ACs has greater resemblance to native cartilage ECM, while cartilage made from MSCs has higher levels of proteins associated with bone formation, negative regulation of cell growth and mitochondrial functionality. Finally, by miRNA sequencing and large-scale target prediction analyses we show that nearly all the differentially expressed proteins are predicted targets of the most differentially expressed canonical miRNAs and isomiRs, suggesting that these miRNAs contribute strongly to the differences in the quality of the cartilage made from the two different types of cells. We conclude that the cells most suitable for tissue engineering of hyaline cartilage are articular chondrocytes.

## 2 Materials and methods

### 2.1 Cell isolation and *in vitro* cell expansion culture

We confirm that all donors provided written, informed consent, and the study was approved by the Regional Committee for Ethics in Medical Research, Southern Norway, approval numbers 2009/742 and 2019/906. We confirm that all research was performed according to relevant guidelines and regulations. No tissues were obtained from prisoners. Articular cartilage was obtained from discarded tissue of three patients with primary OA undergoing knee replacement surgery at the Department of Orthopedic Surgery, Lovisenberg Diakonale Hospital, Oslo, Norway. Cartilage pieces were taken from a part of the surface of the femoral condyle which did not show macroscopic signs of OA. The cartilage tissue was cut and digested as previously described (Fernandes et al., 2013), then resuspended in culture medium: DMEM/F12 GlutaMAX medium (Gibco) supplemented with 10% hPLP, modified after Schallmoser et al. (Schallmoser and Strunk, 2009), 2 IU/mL heparin (Wockhardt), 100 units/mL penicillin and 100 µg/mL streptomycin (P/S) (Sigma-Aldrich), 2.5 µg/mL amphotericin B (Sigma-Aldrich), and 10 ng/mL bFGF (Gibco). The culture medium was changed every 3–4 days. Amphotericin B was discontinued after 1 week. Cells were cultured until passage 2-3.

### 2.2 Isolation and culture of bone marrow mesenchymal stromal cells

BM aspirates were obtained from the iliac crest of three healthy donors as previously described (Shahdadfar et al., 2005) and modified after Jakobsen et al. (Jakobsen et al., 2014) In brief, BM aspirates were obtained in heparin-coated syringes, immediately diluted 1:4 with DMEM-F12 (Gibco) and centrifuged on a density gradient (Lymphoprep, Axis Shield). The mononuclear layer was washed twice and seeded into 175 cm^2^-culture flasks (Nunc). Cells were allowed to adhere for 72 h before the first medium change. The medium was then changed every 3–4 days. MSCs were further cultured in the same culture medium as described for the chondrocytes. Cells were cultured until passage 3.

### 2.3 Cartilage disc preparation

Chondrocytes and MSC were differentiated using the same chondrogenic differentiation medium (CDM): DMEM/F12 GlutaMAX supplemented with 10 ng/mL transforming growth factor β1 (TGFβ1) (R&D systems), 500 ng/mL bone morphogenetic protein 2 (BMP2) (InductOs), 0.1 µM dexamethasone (DexaGalen, GALENpharma), 1% insulin-transferrin-sodium selenite media supplement (Sigma-Aldrich), 0.1 mM ascorbic acid 2-phosphate (Sigma-Aldrich), 1.25 mg/mL human serum albumin (Octapharma), 4.5 g/L glucose (B. Braun), 1 mM sodium pyruvate (Gibco), 40 µg/mL proline (Sigma-Aldrich) and P/S.

For cartilage disc preparation, 500,000 cells were resuspended in 150 µL of CDM and seeded in 6.5 mm polycarbonate transwell inserts in 24-well plates (Corning) as described previously (Frerker et al., 2021). The plates were centrifuged for 5 min at 200 g, and 700 µL of CDM was carefully added to the bottom wells. Inserts were transferred from 24-well plates to 6-well plates on day one of differentiation culture using custom-made adaptors, and 5 mL CDM was carefully added to each well. The small volume in the insert top was changed every second day, whereas the medium in the outer wells was changed every 4–5 days. After 10 days, cartilage discs were stripped off the transwell membrane and allowed to float freely in medium in 6-well plates, which were placed on a shaker that rotated at 65 rpm. To strip the discs from the transwell membrane, a sterile blade was used to partially cut around the membrane periphery on the bottom of the insert, trying to keep parts of the membrane attached to the insert. Then the membrane was carefully peeled off using sterile forceps, and the cartilage disc was released into the medium. Cartilage discs were harvested after 4 weeks of chondrogenic differentiation, washed with PBS, and further treated as described below. Determination of wet weight was done, removing excessive fluid by briefly holding the edge of the cartilage disc on a tissue paper before weighing on a fine scale (METTLER TOLEDO; AG204 DeltaRange).

### 2.4 Western blot analysis

Proliferation and apoptosis were investigated by western blot analysis of PCNA and Caspase-3 using monolayer cells and discs from day 1 and day 2. 0.5 × 10^6^ monolayer cells and discs made from 0.5 × 10^6^ cells were lysed in 100 µL 1x Laemmli buffer and boiled at 95°C for 10 min. Samples were stored at −20°C until analysis were performed. 20 µL of cell lysates and 10 µL of Caspase-3 control extracts (#9663; Cell Signalling Technology) were loaded onto 4%–20% gradient polyacrylamide gels (Bio-Rad). Proteins were separated by gel electrophoresis, transferred to PVDF membranes (Bio-Rad) and incubated with appropriate antibodies simultaneously. Prior to adding primary antibodies, membranes were blocked in 5% milk (skim milk powder; MERCK) in TBS-T (TBS, BioRad and Tween 20, Sigma) for 1 h at RT. Primary antibodies were anti-PCNA antibody (ab18197; Abcam; 1:1,000) (Du and Gao, 2021), Caspase-3 antibody (#9662; Cell Signalling Technology; 1:2,000) (Zhao et al., 2021b) and anti-beta actin (ab8226; Abcam; 1:1,000) (Guo et al., 2021). Secondary antibodies were peroxidase anti-mouse IgG (H + L) (PI-2000; Vector Laboratories; 1:2000) and peroxidase anti-rabbit IgG (H + L) (PI-1000; Vector Laboratories; 1:5,000). Precision Protein StrepTactin-HRP conjugate (Bio-Rad; 1:10,000) was added to the secondary antibody solution. Bands were visualized using Luminata classic and Immobilon forte Western HRP substrates (Milipore) and the myECL imager (Thermo Fisher Scientific). Detection of full-length blots is shown in the Supplementary Figures S1–S3. Proteins have been quantified using Image Lab 6.0 (Supplementary Figure S4). One sample of each condition was analysed per donor.

### 2.5 Isolation of total RNA, cDNA synthesis and real-time RT-qPCR

Cartilage discs were snap-frozen in liquid nitrogen and stored at −80°C until processing. Frozen discs were crushed in liquid nitrogen with a pestle and total RNA was isolated according to the protocol of the miRNeasy mini kit (Qiagen). cDNA synthesis and real-time RT-qPCR were performed following protocols from the manufacturer using the High Capacity cDNA Reverse Transcription Kit (Applied Biosystems) and TaqMan 2x universal PCR Master Mix or TaqMan™ Fast Advanced MasterMix (both Applied Biosystems). cDNA samples were probed for genes relevant for cartilage, hypertrophy and ossification using Taqman gene expression assays (Applied Biosystems). Individual samples were run as technical triplicates. Glyceraldehyde 3-phosphate dehydrogenase (*GAPDH*) was used as endogenous control. Results, based on technical triplicates, are shown as expression relative to *GAPDH* using mean values ± SD from the 3 AC donors and the 3 MSC donors. RNA was isolated and analysed from 1 disc per donor.

### 2.6 Immunofluorescence analysis

Cartilage discs were embedded in Frozen Section Medium (Richard-Allan Scientific Neg50, Thermo Scientific) and frozen in dry ice-cooled isopentane. Frozen tissue blocks were stored at −80°C. The samples were cut in 9–10 µm thick sections on a CryoStar™ NX70 Cryostat (Thermo Scientific), mounted on SuperFrost Plus Adhesion slides and stored at −80°C. Sections were immediately post-fixed for 60 s in cold 95% ethanol directly before immunostaining. Sections were immunostained for the presence of ACAN (clone 4F4; Santa Cruz; at 0.1 µg/mL), COL2 (clone II-II6B3-a; DSHB; at 2.45 µg/mL), COL1 (clone EPR7785; Abcam; at 0.8 µg/mL), COL10 (clone X53 diluted 1:200; generous gift from Prof. Klaus von der Mark). Slides were incubated with primary antibodies diluted in 1.25% BSA in PBS at 4°C overnight. Negative controls were made by omitting primary antibodies (Supplementary Figure S5). The secondary antibodies, goat anti-rabbit IgG conjugated to Alexa 488 and goat anti-mouse IgG conjugated to Alexa 594 (Life Technologies), were diluted 1:400. The stained sections were mounted with Fluoroshield (Sigma), containing DAPI for nuclear staining. Samples were analyzed using an upright Nikon Eclipse E600 microscope equipped with an Olympus ColorView III camera. Images were opened in the image processing software Fiji (Schindelin et al., 2012), colour (red, green, blue) was assigned to the images, and images were merged. Thickness of cartilage discs was measured on the stained sections using Fiji. Five measures from representative areas of a section from each donor were taken. Nuclei were counted on the basis of DAPI stained sections prepared for immunofluorescence analysis. 20 equally-sized fields (200 × 200 px) were counted per donor using Fiji. Sections were prepared from 1 disc per donor.

### 2.7 Transmission electron microscopy

Cartilage discs were fixed in a mixture of 2% glutaraldehyde and 0.5% paraformaldehyde in cacodylate buffer for 24 h at 4°C. Afterwards the samples were post-fixed in 2% osmium tetroxide for 2 h at 4°C and further dehydrated, infiltrated and embedded in epoxy resin (Epon). After polymerization, semi-thin sections were cut and stained with toluidine blue in order to localize the region of interest for making ultrathin sections. Epoxy blocks were then trimmed with respect to the structure of interest, and 70 nm ultrathin sections were cut on an ultramicrotome (Leica, UPC6) followed by staining with 4% uranyl acetate in 40% ethanol and Reynolds’ lead citrate. Prepared samples were examined in a transmission electron microscope (Tecnai12, FEI) (Paulsen et al., 2013). Samples were prepared based on 1 disc per donor.

### 2.8 Protein extraction and digestion

The protocol used was modified after Hosseininia et al. (Hosseininia et al., 2019). Cartilage samples crushed in liquid nitrogen were mixed with 200 µL 8 M urea in 50 mM NH_4_HCO_3_, vortexed for 20 s and stored at 4°C for 18 h. Samples were then centrifuged at 14,000 g for 5 min and protein concentration of the supernatants was measured. 100 µg protein in 200 µL supernatant was reduced by addition of 1 µL 0.5 M DTT at 56°C for 30 min. Cysteins were alkylated (2.7 µL 550 mM IAA) before digestion into peptides by 2 µg lysylendopeptidase (Wako) for 2 h at room temperature. Samples were diluted with 50 mM NH_4_HCO_3_ to UREA concentration of 1 M before further digestion with 4 µg trypsin over night at 30°C. Thereafter the digested samples were passed through an ultrafiltration filter, 30 kDa (amicon) in 10 µL aliquots to remove large glycosaminoglycan-containing peptides. Samples were desalted by home-made reversed-phase chromatography using C18 microcolumns prepared by stacking three layers of C18 Empore Extraction Disks (Varian) into 200-μL pipette tips. Purified samples were diluted to a peptide concentration of 0.16 µg/µL and 3 µL was injected into the LC-MS/MS analysis. Proteins were extracted and analysed from 1 disc per donor.

### 2.9 Mass spectrometry and data analysis

Each peptide mixture was separated by Easy nLC1000 nano-LC system connected to a quadrupole—Orbitrap (QExactive Plus) mass spectrometer (ThermoElectron) for data dependent acquisition (top 10 intense peaks for MS/MS). Peptides were separated on a 50 cm EasySpray column (C18, 2 µm beads, 100 Å, 75 μm inner diameter) (Thermo) using a 120 min gradient up to an acetonitrile concentration of 30%. The resulting MS raw files were submitted to the MaxQuant software version 1.6.1.0 for protein identification and label-free quantification. Carbamidomethyl (C) was set as a fixed modification and acetyl (protein N-term), carbamyl (N-term) and oxidation (M) were set as variable modifications. First search peptide tolerance of 20 ppm and main search error 4.5 ppm were used. Trypsin without proline restriction enzyme option was used, with two miscleavages allowed. The minimal unique + razor peptides number was set to 1, and the allowed FDR was 0.01 (1%) for peptide and protein identification. Label-free quantitation was employed with default settings. The Uniprot database with ‘human’ entries (September 2018) was used for the database searches. Perseus software (ver 1.6.1.3) was used for the statistical analysis of the results. In Perseus known contaminants as provided by MaxQuant (Tyanova et al., 2016) and identified in the samples were excluded from further analysis, normalized intensities were log10 transformed and data filtered to include only those proteins that were identified in at least two out three replicates in at least one cell type. After filtering, proteins identified in only one cell type were considered to be the on/off differences. For further comparison analysis, missing values were imputed from normal distribution and *t*-test was performed with permutation based FDR ≤ 0.05 as the criteria.

### 2.10 Targeted proteomics

37 proteins were analyzed by targeted mass spectrometry using parallel reaction monitoring method on an Orbitrap (QExactive HF) mass spectrometry. For each of the proteins, 1-3 unique peptides were selected based on results from data dependent acquisition analysis of the samples above. The analysis was scheduled with a limited number of peptide precursor ions monitored in 10 min retention times windows. Peptides were separated on a 25 cm EasySpray column (C18, 2 µm beads, 100 Å, 75 μm inner diameter) (Thermo) using a 90 min gradient up to an acetonitrile concentration of 30%. Raw data was submitted to MaxQuant for database search using the same settings as above. The resulting msms. txt file was submitted to Skyline (v.19.1) (MacLean et al., 2010) and was compared to the spectral library created from data dependent acquisition analysis to confirm identity. The three most intense y-ions from the parallel reaction monitoring spectra with highest intensity were used for the quantification of the peptides. Skyline is quantifying the fragment ions based on the peak areas.

### 2.11 MiRNA sequencing and analysis

MiRNA was isolated together with RNA (based on 1 disc per donor) using the miRNeasy mini kit (Qiagen) according to the manufacturer’s instructions. Library preparation was performed using the QIAseq miRNA Library Kit (Qiagen) according to manufacturer’s instructions, using 200 ng/sample input and 15 cycles of PCR amplification. Pooled libraries were sequenced on a single run of a NextSeq 500 (Illumina) with 75 cycle high-output reagents. These procedures were performed simultaneously for all samples.

Raw data was trimmed using FAIRPype, an in-house Python pipeline that performs the analysis as a series of steps. Reads were filtered for low quality reads, adapter trimmed, and collapsed to fasta format to reduce mapping time. Reads were mapped using the Bowtie (Langmead et al., 2009) mapping tool version 1.3.0. As the study was focusing on miRNAs and isomiRs, a reference index for read mapping was built based on the 1918 hairpin sequences present in v22.1 of miRBase. Overlapping entries (i.e., entries sharing identical or overlapping sequences with other entries) were removed using a Python script to produce a final set of 1743 unique hairpin sequences (Zhong X. et al., 2019). A Bowtie index was generated using the bowtie-index program and mapping was performed with parameters -v 2. IsomiR read counting was performed using the small RNA analysis pipeline step ‘ParseSAMForMiRNAs’ with the following conditions: 1) the ‘bleed’ was parameter set to 2. i.e., any read that started and ended within ± 2 nt of the specified start and stop position of a miRNA location specified in miRBase v21.1 was retained, 2) isomiRs sharing the same seed region were counted together, as these are likely to produce similar strong repression on the targets. Estimation of differentially expressed features was performed using EdgeR with the classic Single Factor analysis. Full details and links to the software are provided in the Supplementary Material.

Target Prediction was performed using the miRAW (Pla et al., 2018) software package for a subset of predicted up- and downregulated miRNAs, with the miRAWwrapper Python script to generate the shell scripts for batch execution. The Pita model was selected and both canonical (seed region pairing) and non-canonical (extended seed region pairing) were retained. For 3′UTR targets, Ensembl reference annotation Homo_sapiens.GRCh38.102 was used. For genes with multiple annotated 3′UTRs, these were filtered to retain only 1) TSL1 or TSL2 support and (ii) the longest 3′UTR to give a final target set of 19,362 3′UTRs. As all miRNA target prediction tools have sub-optimal performance (i.e., predictions contain many False Positives and False Negatives) a Python script filterMiRAWpredictions.py was used to filter the prediction sets. In this step, predicted targets with an estimated Mean Free Energy (MFE) between the miRNA and the mRNA < −15 or a target prediction probability <0.99995 were removed. Full details and links to the software are provided in the Supplementary Material.

### 2.12 MiRNA cDNA synthesis and real-time RT-qPCR

MiRNA was isolated as described above (based on 1 disc per donor). cDNA synthesis and qRT-PCR were performed following protocols from the manufacturer using the Taqman MicroRNA Reverse Transcription Kit (Thermo Fisher Scientific). 10 ng miRNA in a total volume of 15 µL was reverse transcribed into cDNA. All samples were run in technical triplicates for RT-qPCR with each replicate containing 1 µL cDNA in a total volume of 15 µL. The thermocycling parameters were 95°C for 10 min, followed by 40 cycles of 95°C for 15 s and 60°C for 1 min. U6 was used as endogenous control. Results are shown as expression relative to *U6* using mean values ± SD from the 3 AC donors and the three MSC donors.

### 2.13 Statistics

For determination of wet weight, thickness measurement, nuclei count and RT-qPCR data, differences between groups were analysed using two-tailed Student’s *t*-test. *p* ≤ 0.05 was considered statistically significant. Results are presented as dot plots with mean and SD. For analysis of proteomics data, Student’s *t*-test with permutation-based FDR (FDR ≤ 0.05) for full proteomics and with *p* ≤ 0.05 for targeted proteomics were used to identify differently expressed proteins. All experiments were performed with *N* = 3 for each experimental group and degree of freedom (DF) = 4. For miRNA analysis, EdgeR-based single factor analysis was used for differential expression with correction for multiple testing with a false discovery rate (FDR ≤ 0.05).

## 3 Results

### 3.1 Cartilage discs made from ACs are thicker

**FIGURE 1:**
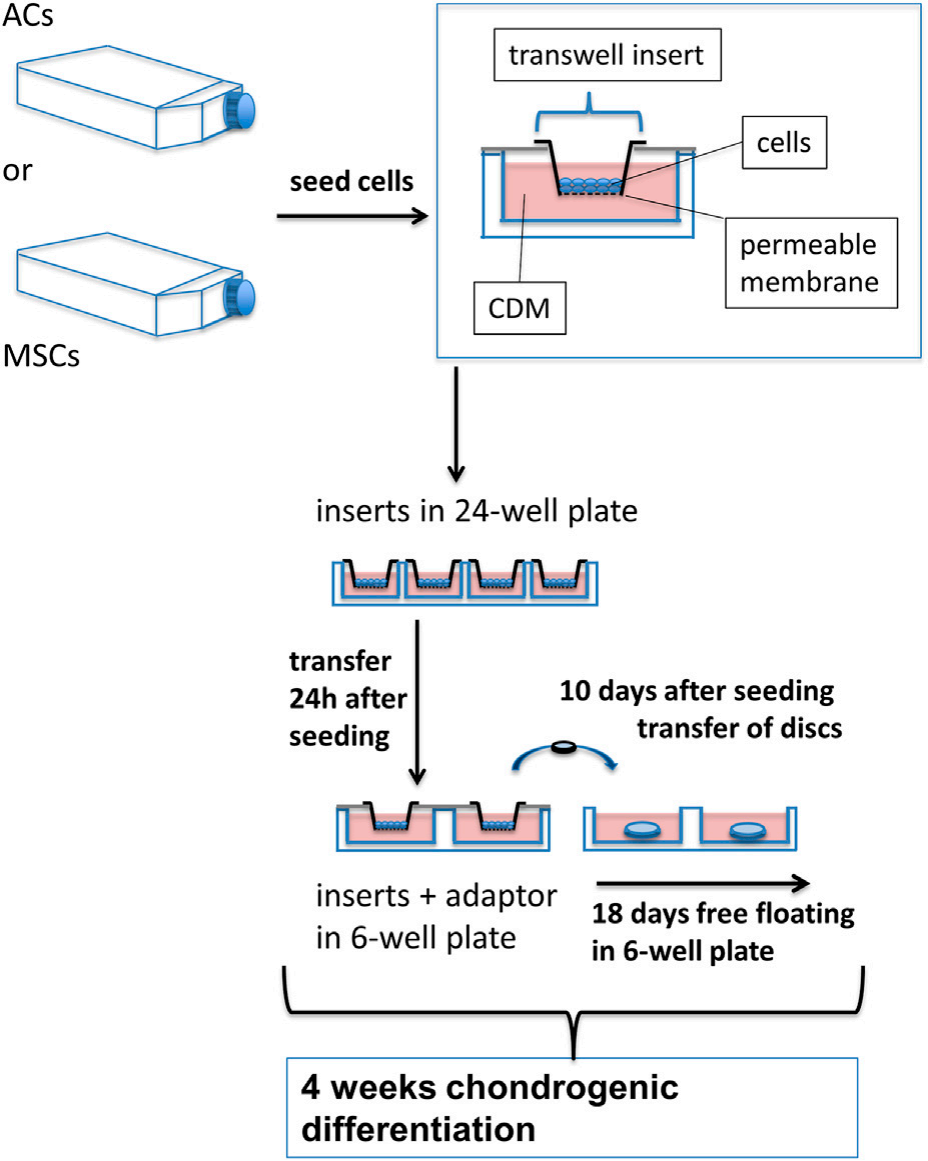
Experimental setup for growing AC and MSC discs. ACs and MSCs from three different donors for each cell type were expanded and then seeded in CDM in transwell inserts, hanging in 24-well plates. 24 h after seeding in CDM, inserts were transferred to 6-well plates, using custom made adaptors. After 10 days of chondrogenic differentiation, AC and MSC discs were released from transwell inserts and kept free-floating in CDM for 18 further days. *N* = 3 for each experimental group.

**FIGURE 2:**
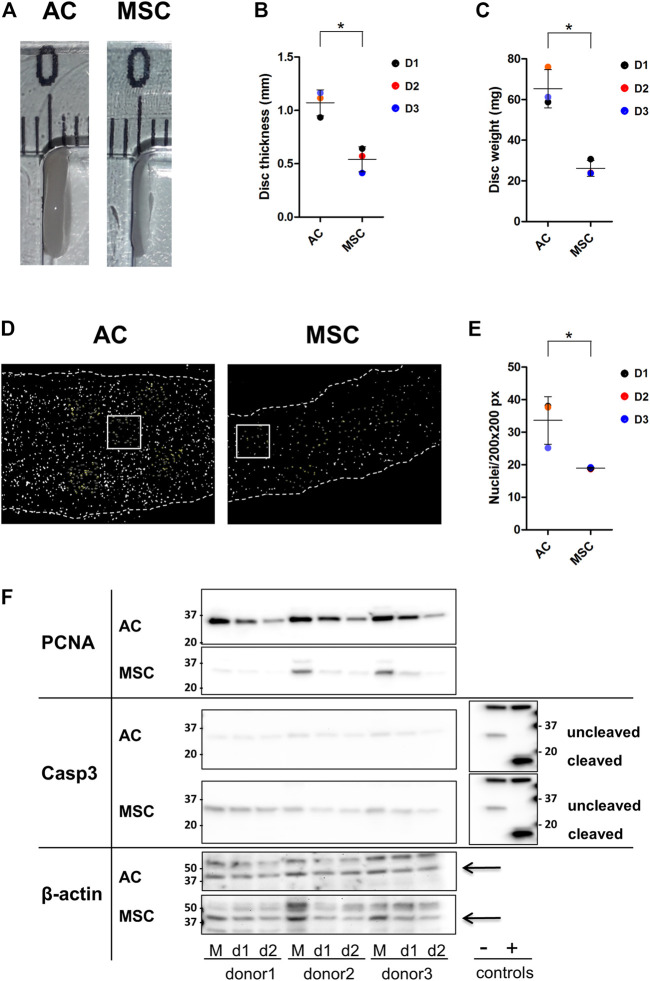
Properties of AC and MSC discs **(A)** Photograph of representative cartilage constructs made by ACs (left) and MSCs (right). **(B)** Thickness (disc height) and **(C)** wet weight of 4-week cartilage discs from ACs and MSCs. Data presented as dot plot diagram with mean and SD; D1-D3 (donor 1–3). **(D)** Nuclei count of 4-week cartilage discs from ACs and MSCs. Nuclei were counted from representative size-defined areas (200 px × 200 px; examples marked with a white box). Nuclei are shown in white. Dashed lines indicate the edges of the cartilage disc section. **(E)** Dot plot diagram of nuclei count with mean and SD; D1-D3 (donor 1–3). **(F)** Western blot analysis. Protein levels of PCNA, Caspase-3 (Casp-3) and cleaved Casp-3 were analysed in monolayer samples (M) and in day-1- (d1) and day-2-discs (d2) of ACs and MSCs. Casp-3 control extracts (Jurkat cell extracts untreated (−) or treated (+) with cytochrome c *in vitro*) showing procaspase-3 (−) and cleaved Casp-3 (+). Beta actin was used as loading control and is indicated by arrows. Samples were derived from the same experiment, AC and MSC samples were loaded on two separate gels, and gels/blots were processed in parallel. Images showing AC and MSC membranes exposed side by side were inverted in Fiji, contrast and brightness were adjusted for beta actin and PCNA. For better comparison, the blot from Casp-3 control extracts has been mirrored and is presented twice. Finally, images were cropped horizontally to show the region of interest. Full-length blots are presented in Supplementary Figure S1 and different exposures of PCNA and Casp-3 are shown in Supplementary Figures S2, S3. Protein quantification data is shown in Supplementary Figure S4. Student’s *t*-test was used for statistics, and *p* ≤ 0.05 was considered significant. *N* = 3 for each experimental group and degrees of freedom (DF) = 4. Wet weight data is based on two discs per donor.

To test which cell type is more suitable for production of cartilage *in vitro*, ACs and MSCs were expanded *in vitro* under identical culture conditions, and then allowed to form discs through differentiation in the presence of identical chondrogenic differentiation cocktails. The culture conditions and differentiation protocol used are, in our hands, the best available for both cell types. The 3D cultures were maintained for 4 weeks (Figure 1). While appearance, such as colour and surface, was similar (Figure 2A), ACs produced on average 2-fold thicker discs compared with MSCs (Figures 2A, B, *p* ≤ 0.05). Consequently, AC discs showed on average 2.5-fold higher wet weight compared with MSC discs (Figure 2C, *p* ≤ 0.05).

The production of thicker and heavier discs by ACs could be due to more cells producing ECM or each cell producing more ECM. Counting cell nuclei per defined area revealed that the density of nuclei in AC discs was higher than in MSC discs (Figures 2D, E) (*p* ≤ 0.05). Thus, although the two populations were seeded on the semipermeable membranes at the same density, at 4 weeks the AC discs contained many more cells. This means that either the ACs proliferated faster than MSCs, or that more MSCs died following establishment in 3D culture. Western blot analysis presented in Figure 2F shows that the band representing the proliferation marker proliferating cell nuclear antigen (PCNA) was much stronger in monolayer AC cultures than in monolayer MSC cultures, and that this difference persisted, with decreasing band strength, through day 2. Further, membranes were tested for caspase-3, which promotes apoptosis when activated by proteolytic processing into p17 and p12 fragments. Uncleaved caspase-3 does not promote apoptosis. The data revealed stronger caspase-3 expression in MSCs compared to ACs. However, neither ACs nor MSCs showed evidence of activated, cleaved caspase-3 (Figure 2F).

These findings indicate that discs made from ACs were thicker because ECM was produced from more cells. There were more cells most likely because ACs proliferated faster after being seeded for disc culture than the MSCs. Neither cell population showed evidence of apoptosis.

### 3.2 Discs made from MSCs show markers of cartilage hypertrophy and ossification

**FIGURE 3:**
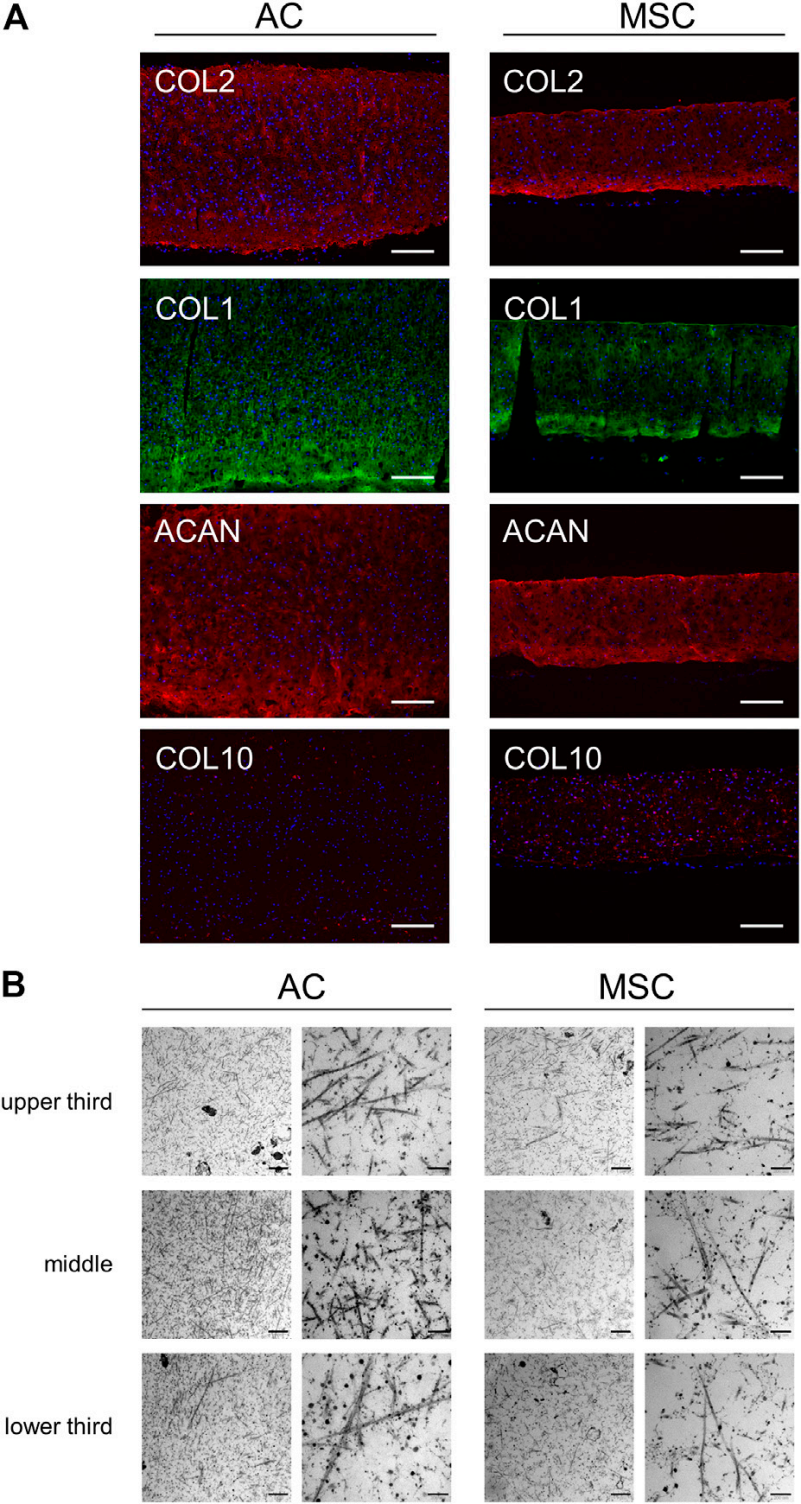
Microscopic analysis of disc sections. **(A)** Immunofluorescence analysis of stained cartilage discs after 4 weeks of differentiation. Sections of AC discs (left) and MSC discs (right) stained for COL2 (red), COL1 (green), ACAN (red) and COL10 (red). Nuclear staining with DAPI is presented in blue colour. Scale bar = 200 µm. One representative donor is shown per condition. **(B)** Transmission electron microscopy of cartilage discs after 4 weeks of differentiation. The presented TEM images from a frontal sample from the middle of the cartilage disc show different sites (upper third, middle, lower third) of the matrix produced by ACs (left) and MSCs (right). Scale bar = 1 µm (left column for each disc type) and 200 nm (right column).

Having determined quantitative differences in cartilage disc production between the two cell populations, we proceeded to investigate qualitative differences between the discs. Immunofluorescence analysis of AC and MSC discs showed similar staining intensities for key chondrogenic molecules type II collagen (COL2), COL1 and aggrecan (ACAN) in discs from both cell types, whereas COL10 expression was clearly stronger in MSC discs. In AC discs, scattered cells close to the edges showed intracellular staining of COL10, while MSC discs showed intracellular as well as diffuse and speckled extracellular staining of COL10 (Figure 3A). Examination of AC and MSC discs using transmission electron microscopy (TEM) showed fibrils of relatively uniform thickness, but with no obvious difference between AC and MSC discs (Figure 3B). Toluidine blue staining (Supplementary Figure S6) indicated an increased concentration of proteoglycans/glycosaminoglycans in the periphery of discs.

**FIGURE 4:**
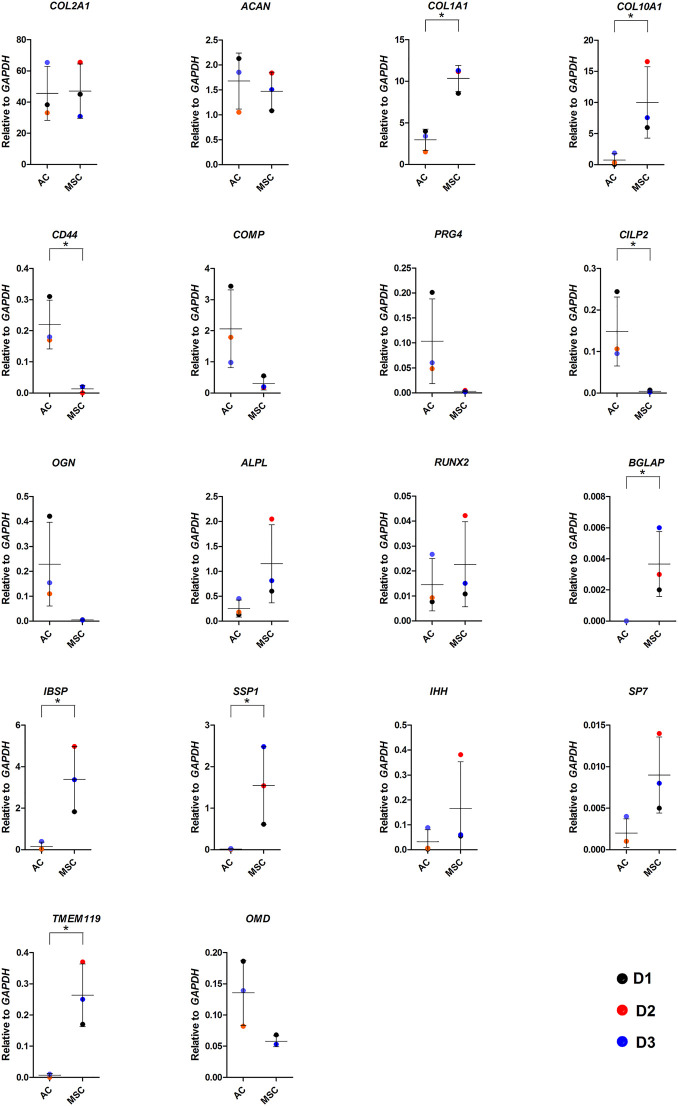
Gene expression in AC and MSC discs. Real-time RT-qPCR analysis of cartilage discs after 4 weeks of differentiation showing cartilage- and hypertrophy/ossification-related markers. Data presented as dot plot diagram with mean and SD; D1-D3 (donor 1–3). Student’s *t*-test was used for statistics, and *p* ≤ 0.05 was considered significant. *N* = 3 for each experimental group and DF = 4.

As COL10 is a marker for hypertrophic cartilage, we proceeded to compare the two types of discs for the expression of genes associated with normal and hypertrophic cartilage and bone formation by RT-qPCR (Figure 4). There were no differences in the expression of hyaline cartilage components *COL2A1* and *ACAN* between discs made from ACs and MSCs, while *COL1A1* levels were significantly higher in MSC discs. As suggested by the immunofluorescence microscopy images, MSC discs revealed significantly higher *COL10A1* expression levels than AC discs. The *COL2A1*/*COL1A1* ratio in AC discs was 15.4 and in MSC discs 4.6, while the *COL2A1*/*COL10A1* ratios were 61.1 and 4.7, respectively. Expression levels of cartilage components *CILP2* and *CD44* were significantly increased in discs made from ACs (*p* ≤ 0.05), whereas levels of *COMP*, *PRG4*, *OGN* and *OMD* were higher, but the differences were not statistically significant. Known drivers of hypertrophy and bone formation such as *BGLAP*, *IBSP*, *SSP1* and *TMEM119* were significantly higher in discs made from MSCs (*p* ≤ 0.05), while *RUNX2*, *IHH*, *ALPL*, *SP7* were higher, but not significantly increased in MSCs (Figure 4). Additional gene expression data is shown in Supplementary Figure S7.

These results suggest that there are neither obvious differences in the major hyaline cartilage components COL2 and ACAN, nor in matrix morphology. However, MSC discs showed increased expression of cartilage hypertrophy and ossification markers.

### 3.3 Proteomics analysis confirms the presence of markers of bone formation in MSC discs

To get a more detailed impression of the differences between discs made from ACs and MSCs we performed high-resolution mass spectrometry-based proteomics analyses on discs differentiated for 4 weeks. More than 3,400 proteins were identified by mass spectrometry data analysis. As a measure for protein abundance, intensity Based Absolute Quantification (iBAQ) (Tyanova et al., 2016) (Supplementary Table S1) showed that most of the highest expressed proteins of one disc type were also among the highest expressed proteins in the other disc type. Of the proteins with aberrant ranking, tenascin (TNC) and cartilage oligomeric matrix protein (COMP) were ranked higher in AC discs, while protein S100-P (S100P) and inorganic pyrophosphatase (PPA1) were ranked higher in the MSC discs.

**FIGURE 5:**
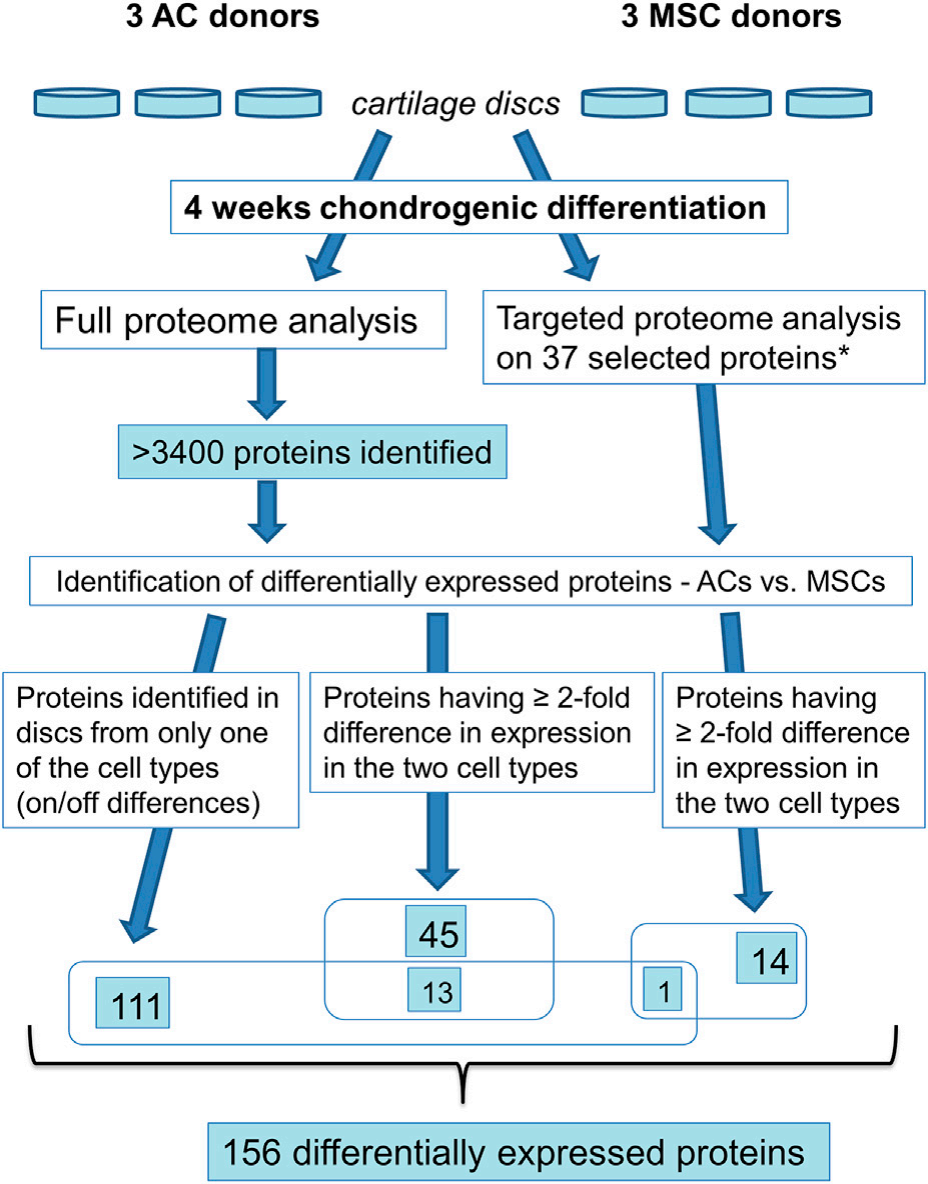
Overview of proteomics analysis. * based on the >3,400 identified proteins 37 proteins were selected for targeted proteome analysis.

We investigated differential expression of proteins using full proteome analysis and targeted proteomics (Figure 5; Supplementary Table S2). First, using label-free quantification (LFQ) (Tyanova et al., 2016) for a comparison of normalised intensity values between samples, we found proteins that were expressed in discs made from one cell population, but not in the other population (on/off proteins). This analysis identified 111 differentially expressed proteins. Further, applying the *t*-test with permutation-based FDR (≤ 0.05, minimum 2-fold difference in expression) as selection criteria we identified 45 significantly differentially expressed proteins. Out of these, 13 proteins were shared with “on/off proteins.” Lastly, we performed targeted mass spectrometry analysis, which has better quantification accuracy than label-free analysis, for some of the proteins that were significantly different in the LFQ analysis based on *p*-value<0.05 only. Selected candidates for this analysis were proteins known to be involved in chondrogenesis, cell proliferation, OA and hypertrophy/ossification processes. Some additional proteins that were not significant based on *p*-value<0.05, but were of particular interest for articular cartilage, namely COL10 and lubricin (PRG4), were also chosen for analysis by targeted proteomics. Altogether 37 proteins were analysed by targeted mass spectrometry, of which 14 proteins were found to be significantly differentially expressed (*p* ≤ 0.05). Out of these, one protein is shared with “on/off proteins.” Thus, altogether 156 proteins were differentially expressed, 72 had higher expression in ACs while 84 were expressed at higher levels in MSCs (Supplementary Table S2).

**FIGURE 6:**
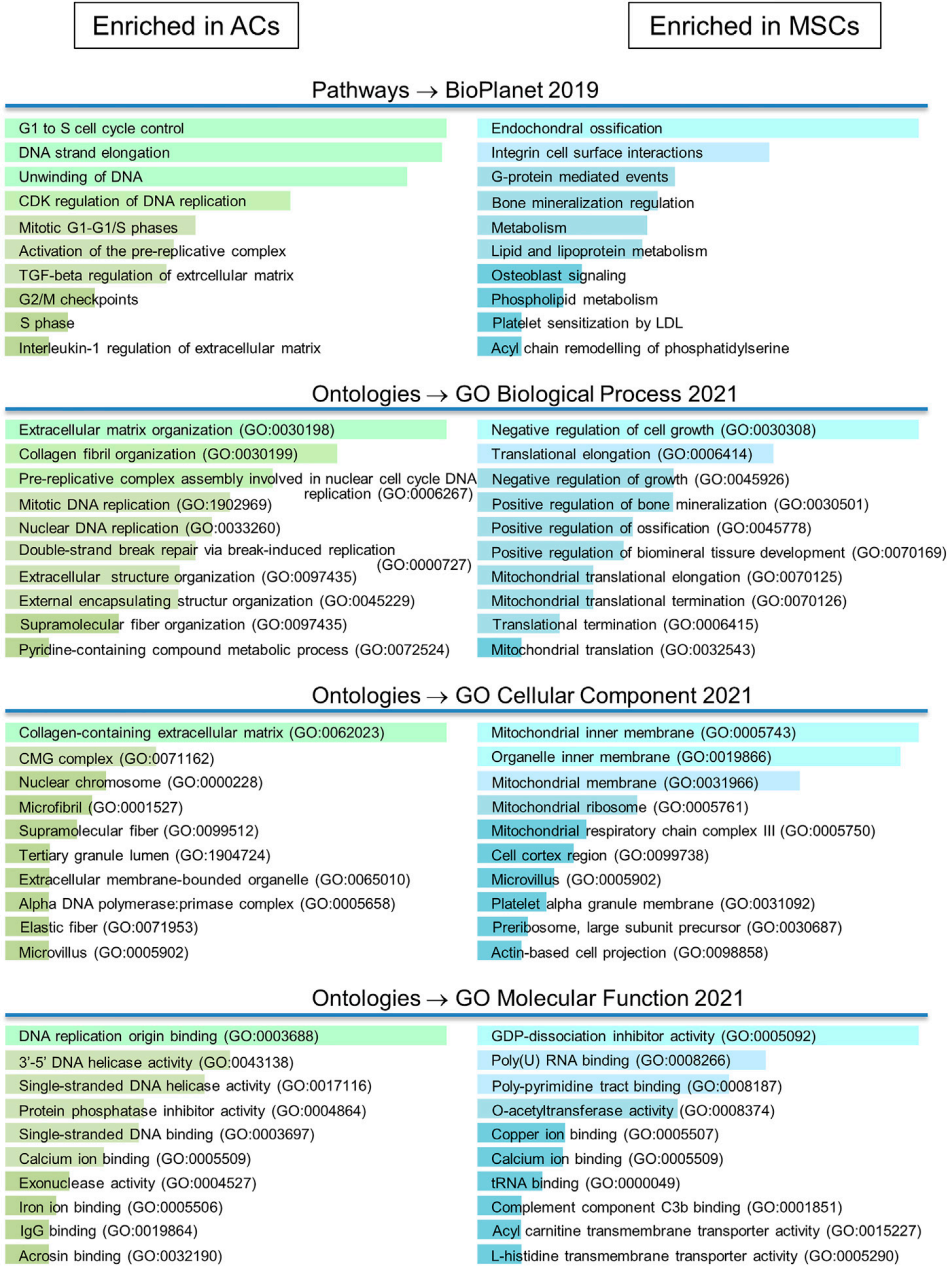
Gene ontology/pathway analysis. Differentially expressed proteins were uploaded in Enrichr. More detailed results from GO/pathway analysis can be found in Supplementary Table S3.

**FIGURE 7:**
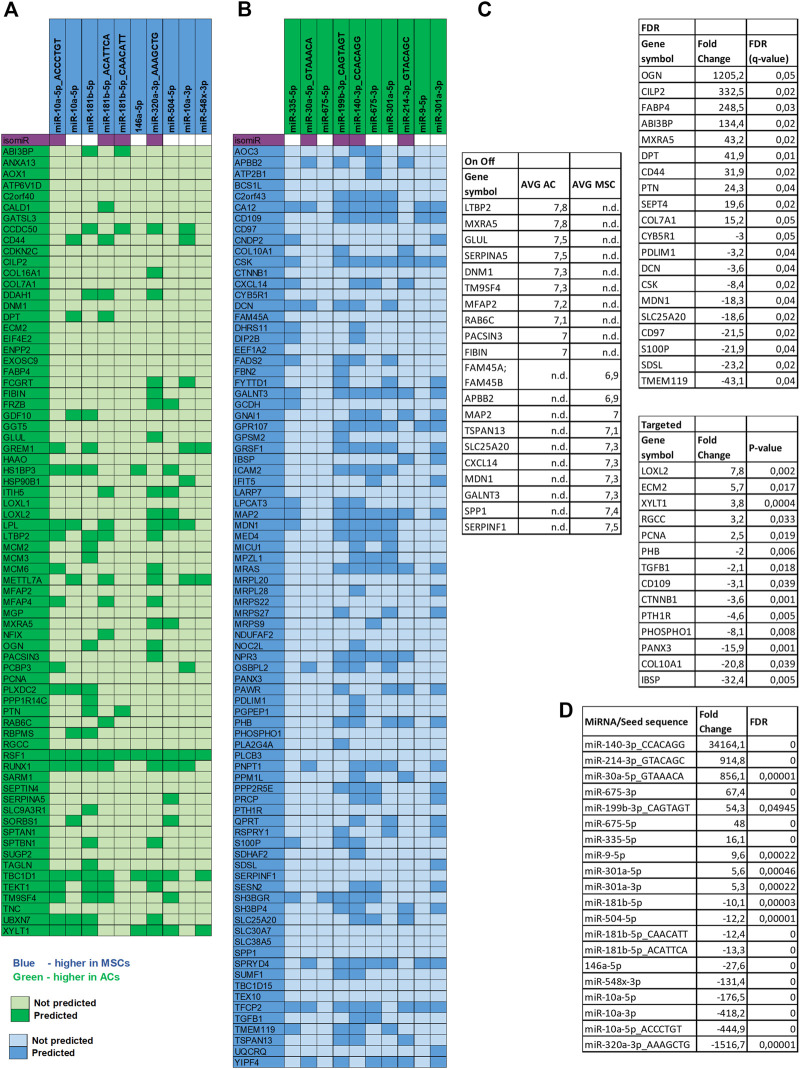
miRNA target analysis. Target mRNA prediction for the top 10 differentially expressed miRNAs in both disc types. **(A)** MiRNAs upregulated in MSCs and proteins downregulated in MSC discs. **(B)** miRNAs upregulated in ACs and proteins downregulated in AC discs. Note: Gene names are used in the figure instead of protein symbols. **(C)** Top 10 DE proteins in AC and in MSC discs of the different approaches of proteomic data analysis are presented. Note: six overlapping proteins of the On Off-list were replaced with the next ranking protein. **(D)** Top 10 DE miRNAs in AC and in MSC discs are presented.

The differentially expressed proteins were uploaded into the analysis program Enrichr (https://maayanlab.cloud/Enrichr/). Here a number of gene ontology (GO) and pathway analyses were applied (Figure 6; Supplementary Table S3). Many proteins upregulated in AC discs were implicated in the organization of the ECM, both collagen fibrils and microfibrils. Of these, gremlin 1 (GREM1) has been found to identify a stem cell with bone, cartilage and stromal potential (Worthley et al., 2015). Furthermore, GREM1 is known to inhibit BMP-2 induced differentiation of osteoblasts and to suppress the production of the hypertrophic marker COL10 (Kišonaitė et al., 2016; Díaz-Payno et al., 2020). Highly differentially expressed mimecan (OGN), dermatopontin (DPT), TNC, COL7A1 and COL16A1, matrix Gla protein (MGP) and lysyl oxidase-like 1 and 2 (LOXL1 and LOXL2) are cartilage ECM constituents, and many of these are associated with collagen cross-linking (Tallheden et al., 2004; Deckx et al., 2016; Mortensen and Karsdal, 2016; Sand and Karsdal, 2016; Lin et al., 2020). Also, highly differentially expressed CD44 is the chondrocyte receptor for hyaluronic acid (HA) (Ishida et al., 1997). The CD44/HA interaction promotes chondrocyte proliferation and is essential for the synthesis of the proteoglycan component of the ECM (Chow et al., 1998). A number of proteins were also involved in the control of the cell cycle. One of these was PCNA (BioPlanet pathway analysis) (Supplementary Table S2; Figure 7C), which was also shown by Western blot to be expressed at higher levels in the very early days of 3D culture (Figure 2F).

The analysis revealed a number of proteins expressed at higher levels in MSC discs that have been reported to be associated with cartilage hypertrophy and bone formation. Beta-catenin (CTNNB1), found under “metabolism” in the pathway analysis, blocks the development of stable chondrocytes from mesenchymal progenitors in favour of pre-osteoblasts (Galea et al., 2021). Transmembrane protein 119 (TMEM119) (Hisa et al., 2011; Tanaka et al., 2012) and fibrillin-2 (FBN2) (Nistala et al., 2010) are important for osteoblast differentiation and bone mineralization. Bone sialoprotein 2 (IBSP) (Ganss et al., 1999; Malaval et al., 2008) and osteopontin (SPP1) (Hunter, 2013; Si et al., 2020) are major non-collagenous structural proteins of bone matrix that bind tightly to hydroxyapatite. Phosphoethanolamine/phosphocholine phosphatase 1 (PHOSPHO1) (Roberts et al., 2004; Roberts et al., 2005) is involved in the generation of inorganic phosphate for bone mineralization, while polypeptide N-acetylgalactosaminyltransferase 3 (GALNT3) (Chefetz et al., 2009) plays a central role in phosphate homeostasis. Alkaline phosphatase (ALPL) (Sharma et al., 2014; Vimalraj, 2020), a key molecule for skeletal mineralization, was expressed more than three times higher in MSC discs (Supplementary Table S1), but this difference was not found to be significant. Finally upregulated protein COL10A1 is a canonical marker for hypertrophic chondrocytes, and it has been strongly argued that *Col10a1+* hypertrophic chondrocytes may become osteoblasts and osteocytes during endochondral bone formation (Yang et al., 2014).

A number of proteins which affect cell growth were upregulated in MSC discs. For instance Sestrin 2 (SESN2) and SH3 domain binding protein 4 (SH3BP4) are both known to negatively regulate cell proliferation through inhibition of the mammalian target of rapamycin complex 1 (Kim et al., 2012; Luo et al., 2018), while disco-interacting protein 2 homolog B (DIP2B) may epigenetically regulate cell proliferation through DNA methylation (Adlat et al., 2020). A number of upregulated proteins in MSC discs were also associated with mitochondrial protein synthesis.

In conclusion, both types of discs contain high levels of proteins known to be essential in normal hyaline cartilage. However, MSC discs contained more proteins known to be associated with bone formation and negative control of the cell cycle.

### 3.4 Evidence that microRNAs regulate the composition of engineered cartilage

Next, we performed small RNA sequencing to investigate if differential expression of miRNAs and associated isomiRs was likely to contribute to differences in cartilage composition (Supplementary Figure S8). 352 miRNAs including isomiRs were detected at greater than 10 reads per million. Out of these, 76 miRNAs were differentially expressed between the two types of discs (fold change ≥2, FDR ≤ 0.05, Supplementary Table S4). 36 miRNAs were at higher levels in ACs and 40 at higher levels in MSC discs. Of all differentially expressed miRNA sequences 40 were 3p strands, 36 were 5p strands and 24 of the 76 sequences were isomiRs. Several of the canonical miRNAs upregulated in ACs, such as miR-675-3p and 5p, miR-335-5p, miR-582-5p and 3p, miR-9-5p, miR-136-3p and miR 495-3p are known to be involved in cartilage biogenesis and disease (Tomé et al., 2011; Díaz-Prado et al., 2012; Chen et al., 2017; Chen et al., 2018; Zhong G. et al., 2019; Chen et al., 2019; Seidl and Murphy, 2019; Wang et al., 2019; Chen et al., 2020; Shen et al., 2020; Zhang et al., 2021). Of the highest differentially expressed canonical miRNAs in MSCs miR-10a-5p, miR-181b, miR-181a, miR-135b-5p and miR-218-5p are known to be involved in osteogenesis, cartilage hypertrophy and degeneration and OA (Bhushan et al., 2013; Song et al., 2013; Gabler et al., 2015; Nakamura et al., 2016; Lu et al., 2017; Ma et al., 2019; Yin et al., 2019; Li et al., 2021). Hardly any of the differentially expressed isomiRs shown in Supplementary Table S4 have known functionality related to cartilage, bone or skeletal diseases.

**FIGURE 8:**
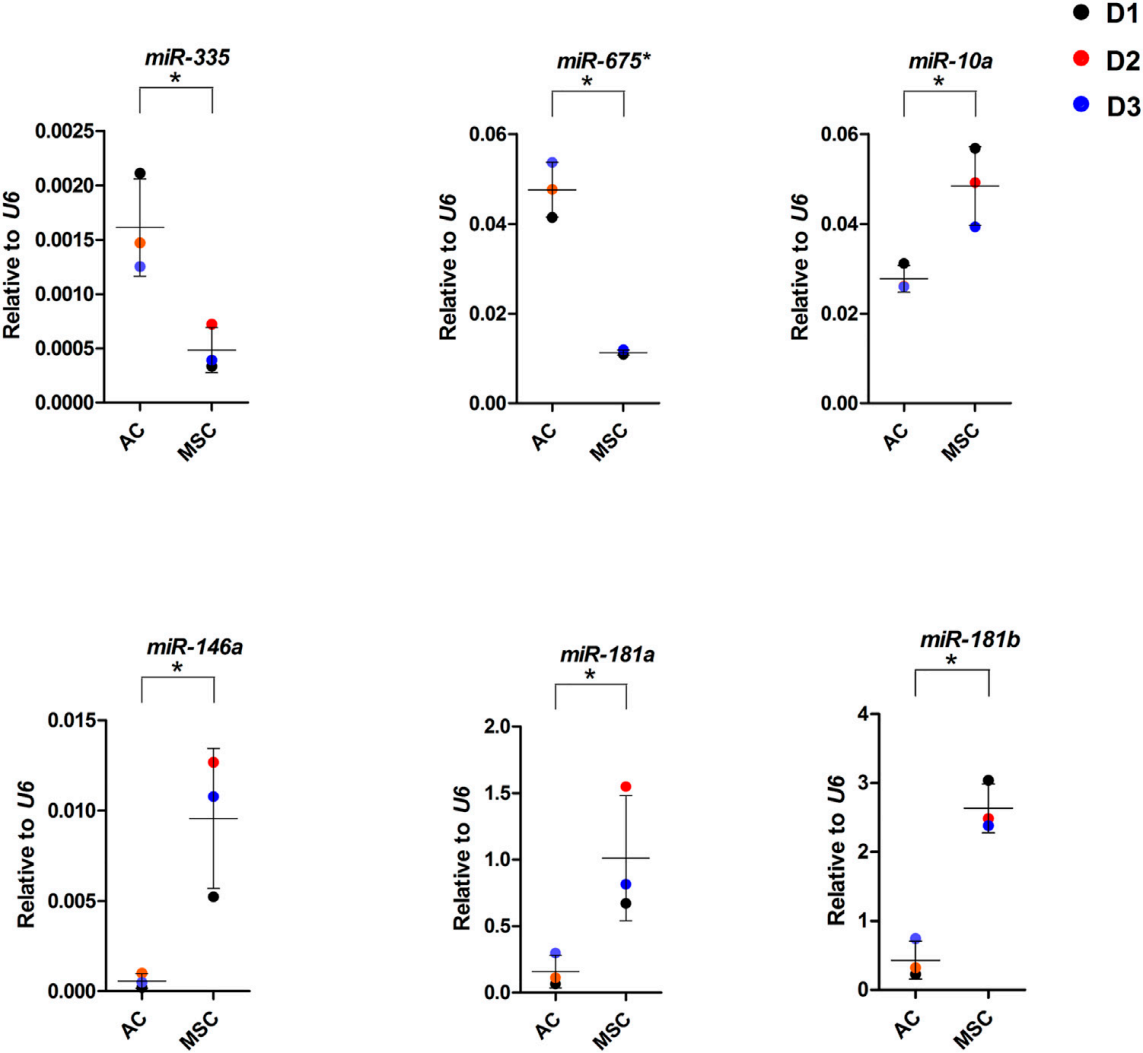
miRNA expression in AC and MSC discs. Real-time RT-qPCR analysis of cartilage discs after 4 weeks of differentiation. MiR-675* = miR-675-3p. Data presented as dot plot diagram with mean and SD; D1-D3 (donor 1–3). Student’s *t*-test was used for statistics, and *p* ≤ 0.05 was considered significant. *N* = 3 for each experimental group and DF = 4.

To further dissect the role played by the differentially expressed miRNAs we performed target mRNA prediction analysis for all the differentially expressed miRNA sequences, and present results for the top 10 differentially expressed miRNAs/isomiRs in both disc types compared with the proteins that were differently expressed by the AC and MSC discs. Figure 7A shows that 46 of the 72 (64%) proteins that had lower expression in MSCs (presented as higher in ACs) were predicted targets of one or more of the top 10 miRNAs that were more highly expressed in MSC discs. Figure 7B shows that 64 of the 84 (76%) proteins that were expressed at lower levels in ACs (thus presented as higher in MSCs) were predicted targets of one or more of the top 10 miRNAs that were higher in AC discs. For additional information, the 10 most differentially expressed proteins from the different approaches of proteomic analysis (based on Supplementary Table S2) and the 10 most differentially expressed miRNAs (based on Supplementary Table S4) are presented for each cell type in Figures 7C, D. Some of these miRNAs were validated using RT-qPCR (Figure 8). These observations strongly suggest that miRNAs contribute to the differences in cartilage phenotypes found in the two disc types.

## 4 Discussion

Transplantation of tissue-engineered cartilage may represent the best treatment solution for focal lesions of articular cartilage. Cartilage discs with a thickness of more than 2 mm may now be made in the laboratory without the support of scaffolds (Frerker et al., 2021). However, an open question has been which cells are able to make the most hyaline-like cartilage ECM, ACs or MSCs. The current study favours the use of ACs as they produce more cartilage when starting with the same amount of cells, and many of the proteins expressed at higher levels in AC discs are components found in normal articular cartilage. Cartilage made from MSCs contains more markers of hypertrophy, bone formation and also expresses proteins related to OA. It appears that miRNAs may have an important role in the regulation of the composition of the ECM produced.

To make the transition from the lab bench to the clinic as smooth as possible we have humanized all our cell cultures. Standard culture medium DMEM/F12 supplemented with 10% human platelet lysate plasma (hPLP) and 10 ng/mL fibroblast growth factor-basic (bFGF) works very well both for ACs and MSCs. Still, we have long known that the population doubling time for MSCs is longer than for ACs. This was also demonstrated by checking levels of PCNA, a processivity factor for DNA polymerase delta and a verified quantification marker for the transition through cell cycle (Zerjatke et al., 2017), which were much higher in ACs than in MSC monolayer cells. This higher level of cell cycle transition in ACs continued through at least 2 days of 3D differentiation culture. In fact, markers of cell cycle, including PCNA, were still elevated in AC discs at 4 weeks. No apoptosis was seen in the beginning of chondrogenic differentiation in these cultures. Although we cannot exclude apoptosis at later time points, we consider the high level of proliferation in ACs to be the reason why AC discs contained more cells than MSC discs at 4 weeks. We believe that the higher number of ECM-producing cells in the AC derived discs was the main reason why these discs were thicker and heavier than the MSC derived discs. However, this conclusion is only valid if the culture conditions and differentiation strategy are the best available for both cell types. They are, in our hands, but we have not compared cell culture and chondrogenic differentiation for all available conditions. Thus, our conclusions on disc thickness should be accepted with the contingency that these results may change if more optimal cell culture and differentiation conditions are identified and used.

Proliferation has previously been shown to be a requirement for chondrogenesis for MSCs in 3D pellet cultures (Dexheimer et al., 2012). This study likened pellet cultures to the embryonic condensation step of mesenchymal progenitors associated with transient withdrawal from the cell cycle until chondroprogenitor cells are established. During embryogenesis the chondroprogenitors then resume cell division while at the same time producing large amounts of ECM. The authors further considered proliferation to be essential for the successful transition of the cells from monolayer to 3D culture, while the second wave of proliferation was directly correlated with ECM synthesis. We have not established two waves of proliferation in our 3D differentiation culture, but we know that the ACs have a more rapid cell cycle when they enter 3D cultures, and we know that they have a higher level of cell cycle markers at 4 weeks. Thus, a closer resemblance to embryonic chondrogenesis may be the main reason why ACs produced more cartilage per initially seeded cell than MSCs.

The fundamental molecular components that make up most of hyaline cartilage ECM were not different between AC and MSC discs. COL2 and ACAN were similar by immunostaining, RT-qPCR and proteomics analysis, and other fibrillar collagens were also expressed at the same level between the disc types. COL1 was expressed at a significantly higher level in MSC discs by RT-qPCR, and was also numerically higher by proteomics analysis, but this difference did not reach significance. COMP and PRG4 were also higher in AC discs by both RT-qPCR and proteomics analysis but, again, the difference did not reach significance. The density and thickness of the collagen fibrils looked the same by TEM. Thus, based on the ECM proteins found in the two disc types, they should probably both be characterized as variants of hyaline cartilage.

There were also important differences in the proteins that made up the two types of discs. Many proteins associated with the synthesis and organisation of the ECM were expressed at higher levels in the AC discs. Some of these proteins are associated with collagen fibril formation (OGN, LOXL1, LOXL2, COL16) (Deckx et al., 2016; Sand and Karsdal, 2016; Lin et al., 2020). While we did not see definite differences in the appearance of the fibrils by electron microscopy, the higher expression of these proteins in the AC discs may impact the quality and life span of the fibrils, properties that would not be revealed by assays employed in this project. Interestingly, many of the proteins highly expressed in the AC discs are known to modify the activity of TGFβ (OGN, TNC, DPT, COL7A1, others) (Okamoto et al., 1999; Carey et al., 2010; Deckx et al., 2016; Martins et al., 2016). TGFβ was used in our chondrogenic differentiation medium, both for AC and MSC discs. The higher expression of these proteins may conceivably be responsible, at least in part, for the differences observed between AC and MSC discs following chondrogenic differentiation, which was largely driven by identical concentrations of TGFβ.

A number of proteins associated with cartilage hypertrophy and/or ossification were more abundant in MSC discs. This could conceivably be due to the addition of BMP2 at 500 ng/mL, a somewhat higher dose than that used by some other researchers in the field. We have found that the addition of BMP2 to the chondrogenic differentiation cocktail is essential for the robust formation of cartilage discs (Frerker et al., 2021), and chose this dose based on the landmark paper by the Prockop group (Sekiya et al., 2005), where this dose was chosen based on dose/response experiments. They noted increase in the mRNA levels for hypertrophic markers *COL10A1*, *PTHLH*, *IBSP* and *OMD* in the course of differentiation, but felt that this was an *in vitro* phenomenon typical of chondrogenic differentiation of MSCs, and noted that their MSCs had retained a chondrogenic phenotype through 42 days of differentiation. In a factorial quality-by-design study we examined the effect of adding BMP2 500 ng/mL to a chondrogenic differentiation cocktail containing TGFβ1 and dexamethasone at the same doses as used in the present study for the gene expression during chondrogenic differentiation of MSCs (Jakobsen et al., 2014). We did not find differential expression of any genes associated with hypertrophic chondrogenesis. Finally, if this concentration of BMP2 should invariably lead to the development of hypertrophic chondrocytes one would expect this to happen equally in both cell types used in the present study, but we show an overexpression of hypertrophic proteins in the MSC-derived discs only.

Of the differentially expressed proteins COL10 is known to be an important marker for hypertrophic cartilage (Mueller and Tuan, 2008), although its biological role is not fully known. CTNNB1 induces osteogenic rather than chondrogenic differentiation in mesenchymal progenitors (Galea et al., 2021). TMEM119, FBN2, IBSP and SPP1 are involved in osteoblast differentiation, synthesis and remodelling of bone ECM and matrix calcification (Ganss et al., 1999; Malaval et al., 2008; Nistala et al., 2010; Hisa et al., 2011; Tanaka et al., 2012; Hunter, 2013; Si et al., 2020), while PHOSPHO1 (Roberts et al., 2004; Roberts et al., 2005) and GALNT3 (Chefetz et al., 2009) are involved in the generation of inorganic phosphate for bone mineralization or play a central role in phosphate homeostasis, respectively. Taken together, the overexpression of these and several other proteins in MSC discs suggests an ongoing process in these discs which resembles the endochondral ossification process during embryogenesis (Galea et al., 2021).

We present two types of evidence to suggest that miRNAs are important for the composition of the cartilage engineered from the two types of cells. First, out of the 20 most differentially expressed miRNAs expressed at higher levels in the AC discs, many are known from the literature to stimulate chondrogenesis or have a favourable function relative to OA or hypertrophy/ossification. For instance overexpression of miR-675-3p has been found to inhibit apoptosis and cartilage matrix degradation and promote cell proliferation in human chondrocytes (Shen et al., 2020). The 3′ arm of miR-675 was found to be the only functional arm of miR-675 in ACs, and was found to downregulate cartilage degrading enzymes matrix metalloprotein 1 (MMP1) and MMP13 (Seidl and Murphy, 2019). MiR-335-5p has been shown to inhibit adipogenic and osteogenic differentiation of hMSCs (Tomé et al., 2011). MiR-582-5p has been shown to suppress osteogenic differentiation of MSCs and to decrease *ALPL* and *RUNX2* mRNA levels (Wang et al., 2019). MiR-582-3p has been shown to be upregulated in normal relative to OA chondrocytes in aggregate cultures (Díaz-Prado et al., 2012), and miR-9-5p was reported to inhibit apoptosis in chondrocytes (Chen et al., 2019). In contrast, of the 20 most differentially expressed miRNAs expressed at higher levels in the MSC discs, many are known to be associated with cartilage hypertrophy/ossification and disease. Among the highest differentially expressed canonical miRNAs in MSCs was miR-10a-5p, which has been found to be upregulated in OA cartilage and to promote progression of OA (Ma et al., 2019), and miR-10a-3p, whose overexpression has been shown to improve cartilage degeneration in a knee OA rat model (Li et al., 2021). MiR-181b-5p and -3p as well as miR181a-5p and -3p are among the highly differentially expressed miRNAs in MSC discs. In general, miR-181b has been suggested to be a negative regulator of cartilage development, to be upregulated in OA chondrocytes, and its attenuation induced COL2 expression and reduced MMP-13 expression in chondroblasts and articular chondrocytes (Song et al., 2013). For miR-181a roles in osteoblastic differentiation (Bhushan et al., 2013) and hypertrophy were described (Gabler et al., 2015). In accordance with this miR-181a-5p has been shown to be upregulated in OA cartilage and hypertrophic chondrocytes, and to promote cartilage degeneration and osteoblastic differentiation (Nakamura et al., 2016). Overexpression of miR-135b-5p has been shown to promote osteogenic differentiation and calcification (Yin et al., 2019). MiR-218-5p was reported to be upregulated in moderate and severe OA and its downregulation was shown to promote matrix synthesis, cell proliferation, and to inhibit apoptosis (Lu et al., 2017).

Second, we found that 64% and 76% of all the downregulated proteins in MSC and AC discs, respectively, had mRNAs that were predicted targets of one or more of the top 10 differentially upregulated miRNAs in each of the disc types. One example is OGN, the most highly overexpressed protein in AC discs. We found OGN to be a predicted target of miR-320a-3p isomiR, the most highly differentially expressed miRNA sequence and miR-181b-5p, also highly differentially expressed. Both of these miRNA sequences were upregulated in MSC, or downregulated in AC discs. One possible chain of events may be that, either as a result of pre-existing differential expression or as a result of differential effect of the chondrogenic differentiation procedure, these miRNAs are downregulated in AC discs. When the miRNAs are downregulated, their predicted target, OGN, will be upregulated. The same mechanism may apply to all the miRNA/predicted target pairs presented in Figure 7. However, as we have compared AC and MSC discs without knowledge of the miRNA expression in the undifferentiated cells, we do not know whether the primary process is upregulation of a miRNA in one cell type or downregulation of the miRNA in the other.

IsomiRs are still novel, and most of the differentially expressed isomiRs have not been studied previously. Therefore we can only refer to the functions of some of the corresponding canonical miRNA, as canonical miRNAs and their isomiRs often regulate the same pathways (Cloonan et al., 2011). MiR-214-3p, miR-30a-5p and miR-199b-3p are among the highest expressed isomiRs in ACs. The canonical sequence of miR-214-3p has been shown to have cartilage- and chondroprotective effects and to be downregulated in OA cartilage (Cao et al., 2021). Canonical miR-199b-3p has been reported to promote chondrocyte proliferation and to inhibit apoptosis (Gu et al., 2021). The sequence with the highest upregulation in our AC discs is an isomiR of miR-140-3p with a single nt deletion in the 5′ end and thus presents a seed sequence that is distinct from the canonical miR-140-3p. This isomiR has been previously studied in our group and found to possess strong and broad anti-inflammatory effects *in vitro* (Al-Modawi et al., 2021). The functionality of more of the isomiRs presented here will surely be revealed, but these studies are made difficult by the sequence similarity between canonical miRNAs and their isomiRs. The analyses presented here, where targets among differentially synthesised proteins were identified for all the differentially expressed miRNAs or isomiRs, are the first to show that miRNA-driven regulatory loops are of major importance for *in vitro* chondrogenesis.

In our analysis of the possible role of differentially expressed miRNAs we have not considered the absolute expression levels of the miRNAs. Thus, the 10 most differentially expressed miRNAs/isomiRs in each of the disc types, used for target prediction among the differentially expressed proteins, were ranked by their fold change values. This is because, to the best of our knowledge, it is not known whether a change from no to low level expression is more or less important than a change from a high to an even higher level expression. When this knowledge becomes available it may change some of the emphasis given to some of the miRNAs presented here, but we believe it is unlikely to alter the larger picture.

In conclusion, we recommend that ACs should be used by scientists wishing to make cartilage in the laboratory because ACs make more cartilage per initially seeded cell. This is most likely because the ACs have a faster proliferation rate on entering the 3D differentiation culture, perhaps also because ACs to a greater extent may enter a second wave of proliferation associated with increased ECM production. ACs and MSCs make similar amounts of the major molecular components of hyaline cartilage, but many of the lesser components are expressed at higher levels in the AC discs. Finally, ACs make smaller amounts of many proteins associated with hypertrophy and ossification, and this is likely to be due in part to the overexpression of miRNAs targeting these proteins.

## Data Availability

The datasets presented in this study can be found in the article/Supplementary Material. The mass spectrometry proteomics data have been deposited to the ProteomeXchange Consortium via the PRIDE (Perez-Riverol et al., 2019) partner repository with the dataset identifier PXD029434. MicroRNA sequencing raw data are deposited in the SRA database, submission number SUB10450856, BioProject ID: PRJNA779688. The following are the web links for the GitHub repositories: https://github.com/CBGOUS/frekner_SmallRNAPipeline; https://github.com/CBGOUS/frekner_bagcore; https://github.com/CBGOUS/frerkner_data. The miRNA sequencing raw data was analysed using our FAIRPype analysis pipeline, downloadable as a jar file, together with configuration files, from https://github.com/CBGOUS/frekner_SmallRNAPipeline, together with all Python scripts listed in supplementary materials and methods for miRNA data.
