# Supplementary material for: Comparison between articular chondrocytes and mesenchymal stromal cells for the production of articular cartilage implants
Source: Front Bioeng Biotechnol. 2023 Feb 21;11:1116513. doi: 10.3389/fbioe.2023.1116513 (PMC9989206; doi:10.3389/fbioe.2023.1116513)
Supplement: Supplementary file 3 [file DataSheet1.PDF]

Content description

Supplementary Figure S1: Western blot analysis – full length blots

Supplementary Figure S2: Western blot analysis – PCNA – different exposures

Supplementary Figure S3: Western blot analysis – Caspase-3 – different exposures

Supplementary Figure S4: Western blot quantification

Supplementary Figure S5: IHC/IF Negative control – secondary antibody

Supplementary Figure S6: Toluidine blue staining

Supplementary Figure S7: Gene expression in AC and MSC discs

Supplementary Figure S8: Overview miRNA analysis

Supplementary Material and Methods (miRNA analysis)

Supplementary Statistics information

Supplementary Figure S1

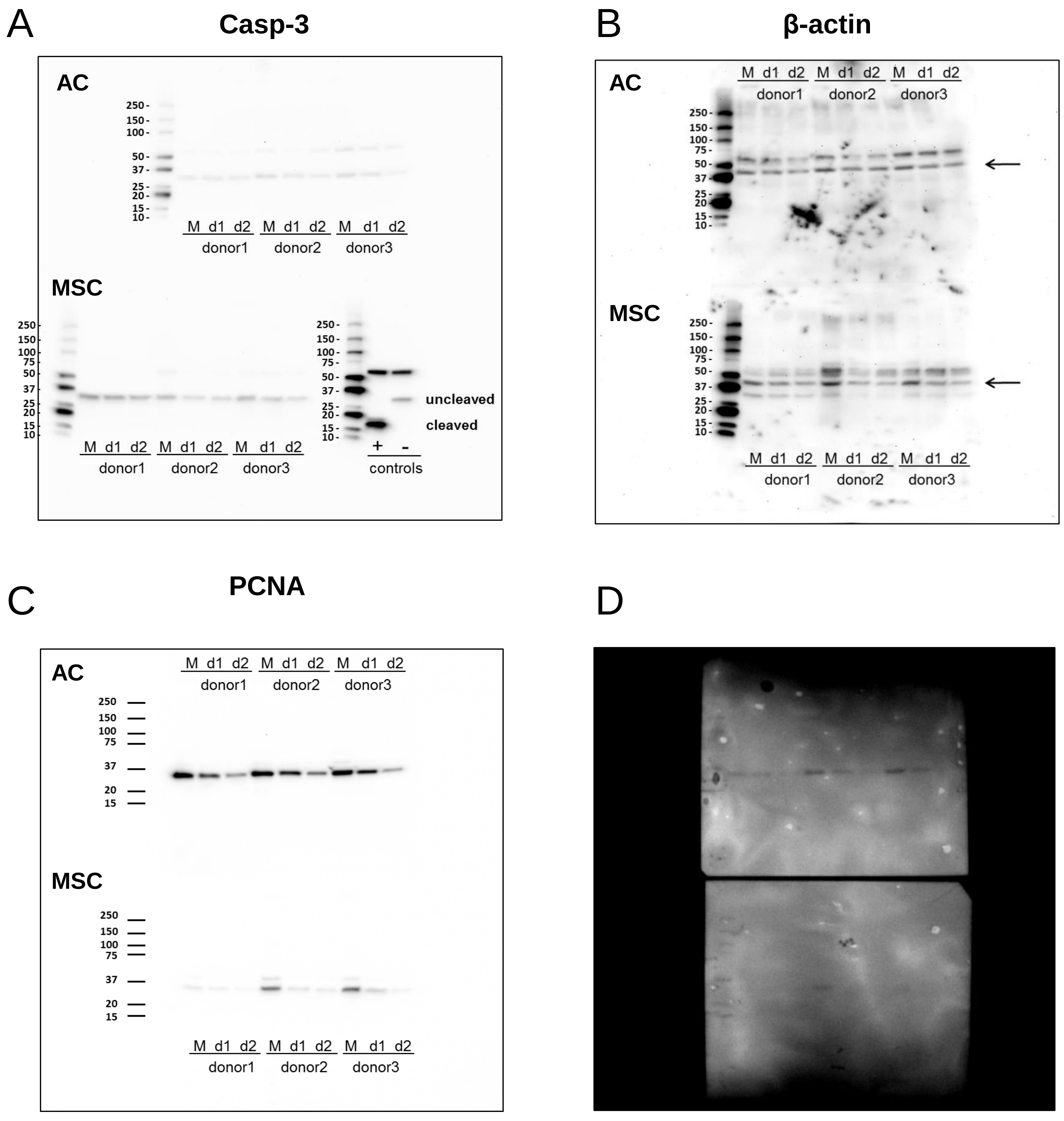

Supplementary Figure S1:
Western blot analysis. Full-length blots tested for **(A)** Caspase-3 (Casp-3) and cleaved Casp-3, **(B)** beta actin as loading control (band indicated by arrows), and **(C)** PCNA in monolayer samples (M) and in day-1- (d1) and day-2-discs (d2) of ACs and MSCs.
(D) Photograph of the PCNA membranes acquired during chemiluminescent exposure showing the pre-stained marker; contrast/brightness was adjusted in Fiji.

Supplementary Figure S2

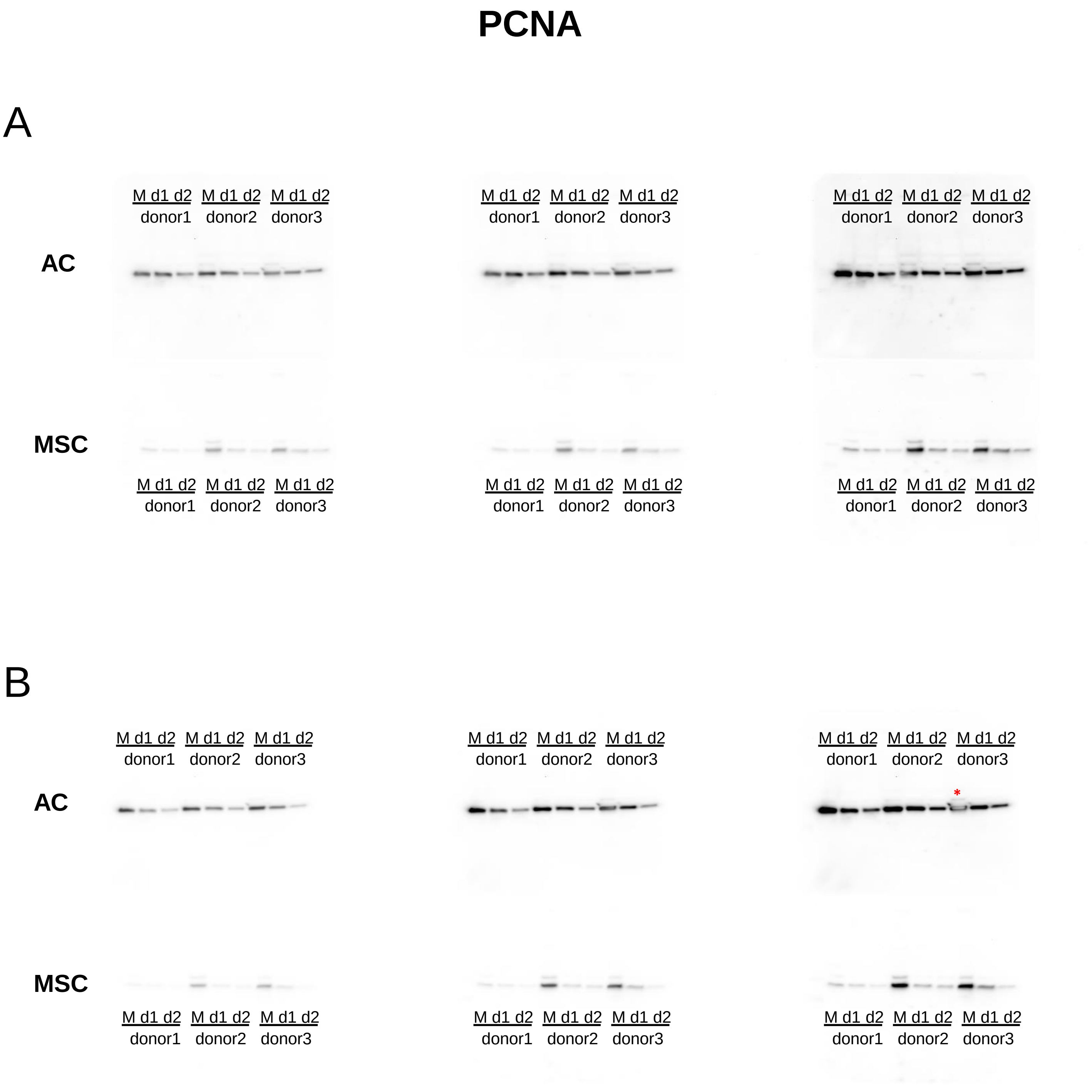

Supplementary Figure S2:
Western blot analysis. Full-length blots tested for PCNA in monolayer samples (M) and in day-1- (d1) and day-2-discs (d2) of ACs and MSCs. The two membranes were not cut and exposed simultaneously. Variable times of chemiluminescent exposure using 2 different substrates are shown in **(A)** (Milipore, Luminata classico Western HRP substrate) and **(B)** (Milipore, Immobilon forte Western HRP substrate). Images were inverted using myImageAnalysis software (Thermo Scientific, v1.1).
* Example of overexposure.

Supplementary Figure S3

Caspase-3/cleaved caspase-3

A

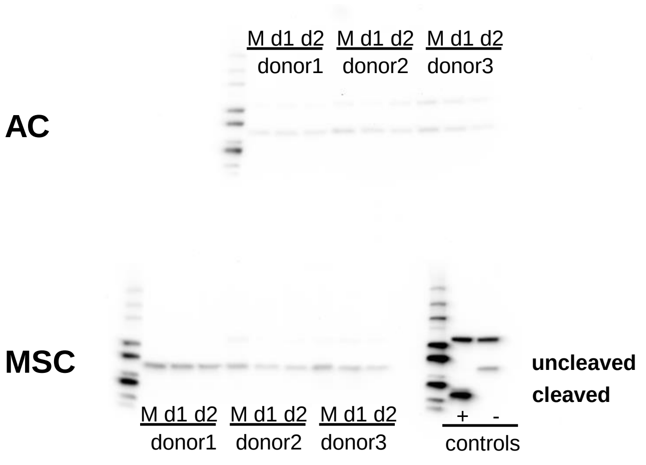

C

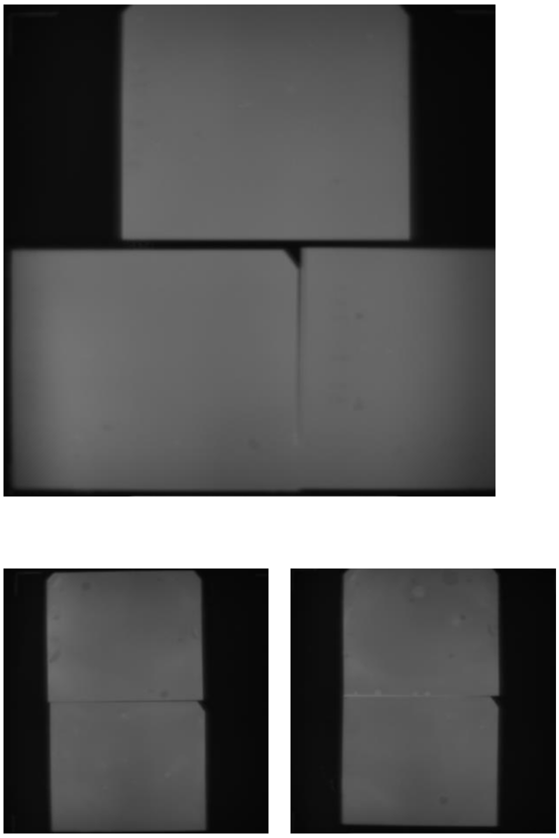

B

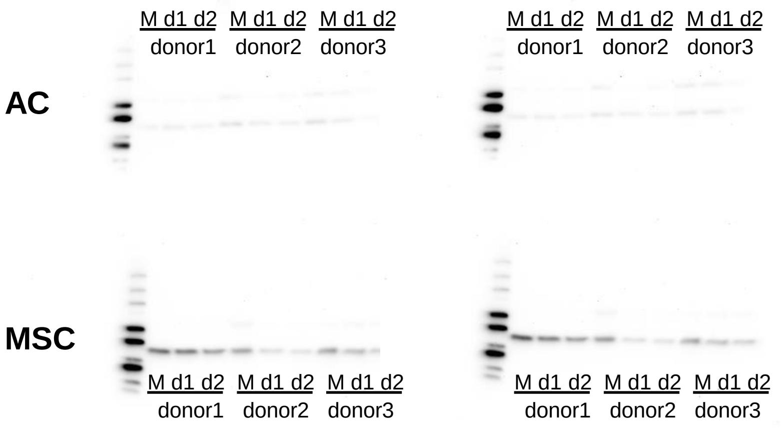

Supplementary Figure S3:
Western blot analysis. Full-length blots tested for caspase-3 (Casp-3)/cleaved Casp-3 in monolayer samples (M) and in day-1- (d1) and day-2-discs (d2) of ACs and MSCs and in Casp-3 control extracts.
(A) AC and MSC membranes were exposed simultaneously with the membrane of Casp-3 control extracts (Jurkat cell extracts untreated (-) or treated (+) with cytochrome c *in vitro*) showing procaspase-3 (-) and cleaved Casp-3 (+).
(B) Variable times of chemiluminescent exposure for AC and MSC membranes.
(C) Photographs of Casp-3 membranes acquired during chemiluminescent exposure showing that membranes were not cut and exposed simultaneously. Images were inverted using myImageAnalysis software (Thermo Scientific, v1.1).

Supplementary Figure S4

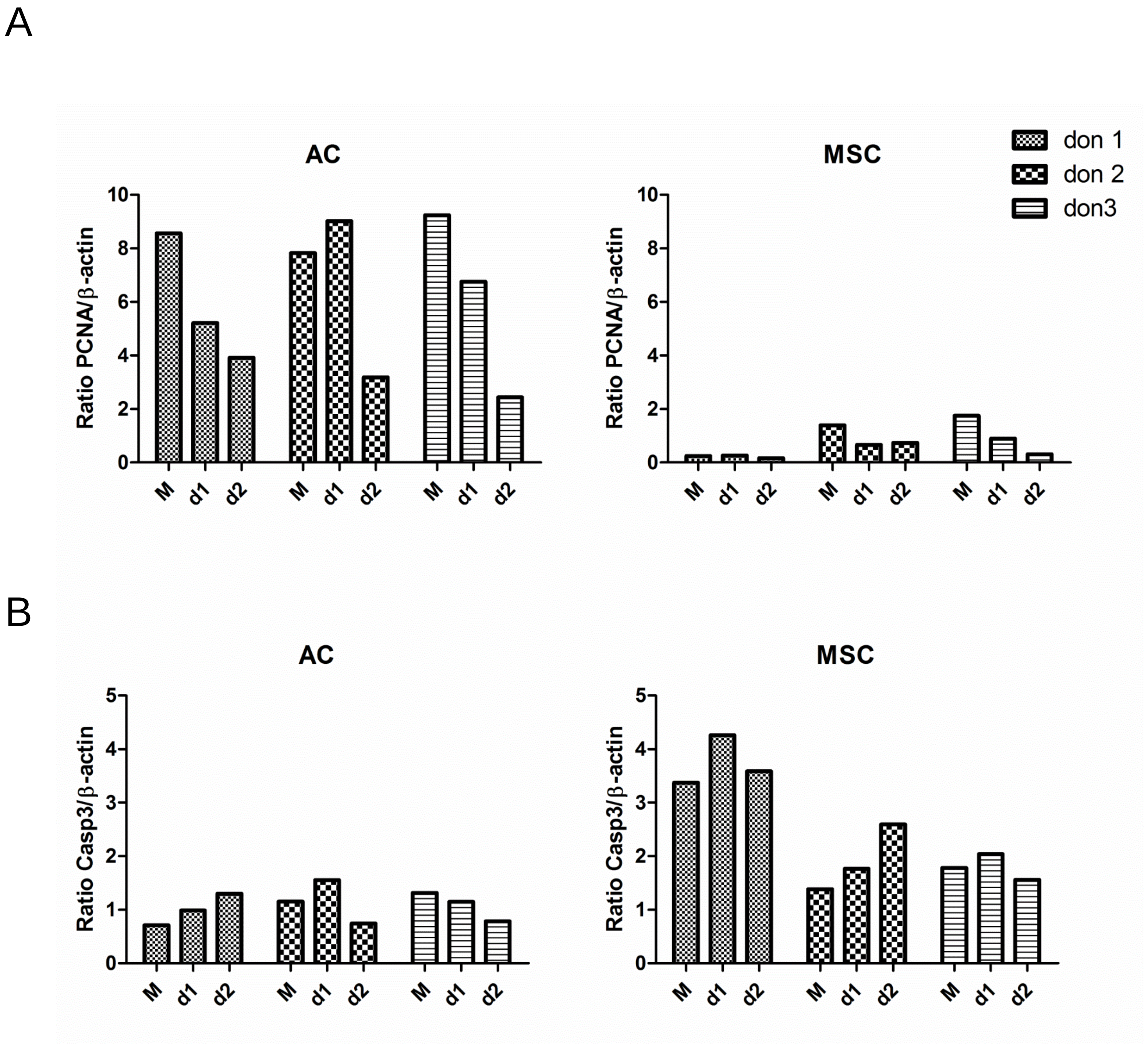

Supplementary Figure S4:
Western blot quantification. Quantification of raw data using Image Lab 6.0 presenting the ratio of **(A)** PCNA vs. β-actin and **(B)** Caspase 3 (Casp3) vs. β-actin for AC and MSC membranes.

Supplementary Figure S5

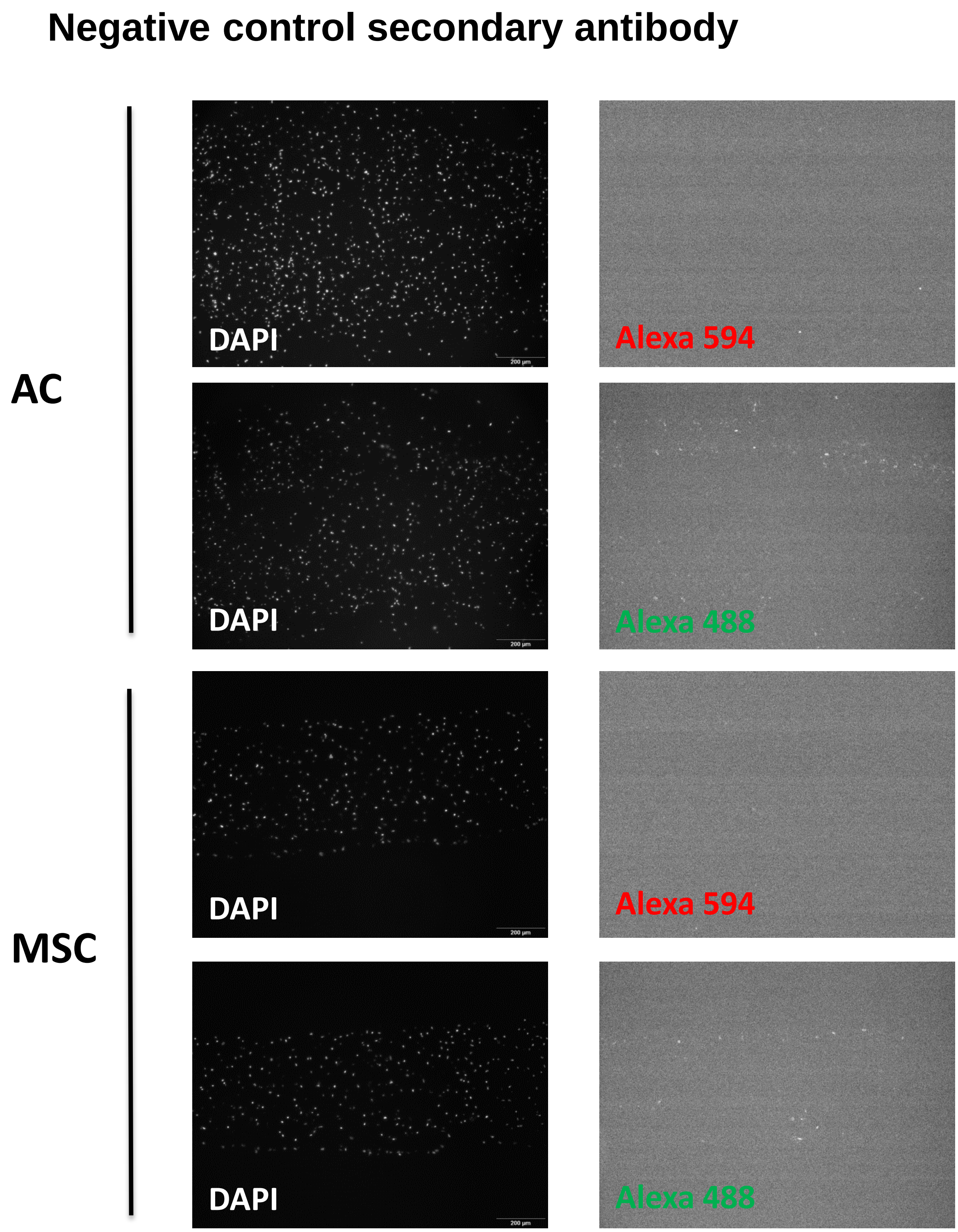

Supplementary Figure S5:
IHC/IF - Negative control for secondary antibody. Negative controls were made by omitting primary antibodies on sections of AC (top) and MSC discs (bottom).
The secondary antibodies were goat anti-rabbit IgG conjugated to Alexa 488 and goat anti-mouse IgG conjugated to Alexa 594. The panel to the left shows DAPI staining whereas the right panel shows lack of proper signals. Faint background signals seen for Alexa 488 do not have an impact on COL1 matrix staining.

Supplementary Figure S6

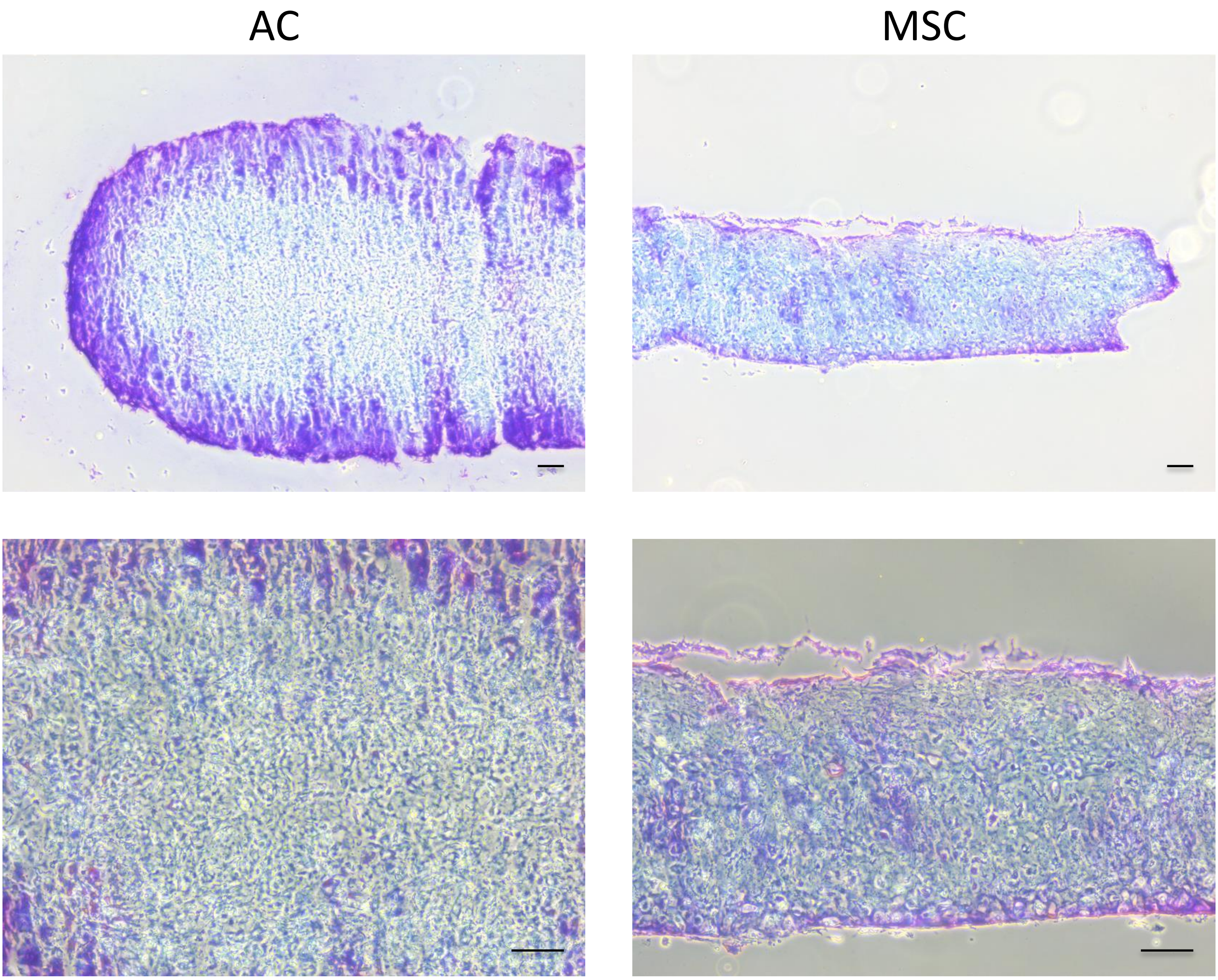

Supplementary Figure S6:
IHC – Toluidine blue staining. Frozen sections fixed in EtOH (95%, 45 s) and immediately stained in 0,1% toluidine blue solution (15 s). Images taken with different magnifications are shown for ACs (left) and MSC (right). Brightness and contrast have been adjusted equally for AC and MSC images of the lower panel. Scale bar = 100 µm

Supplementary Figure S7

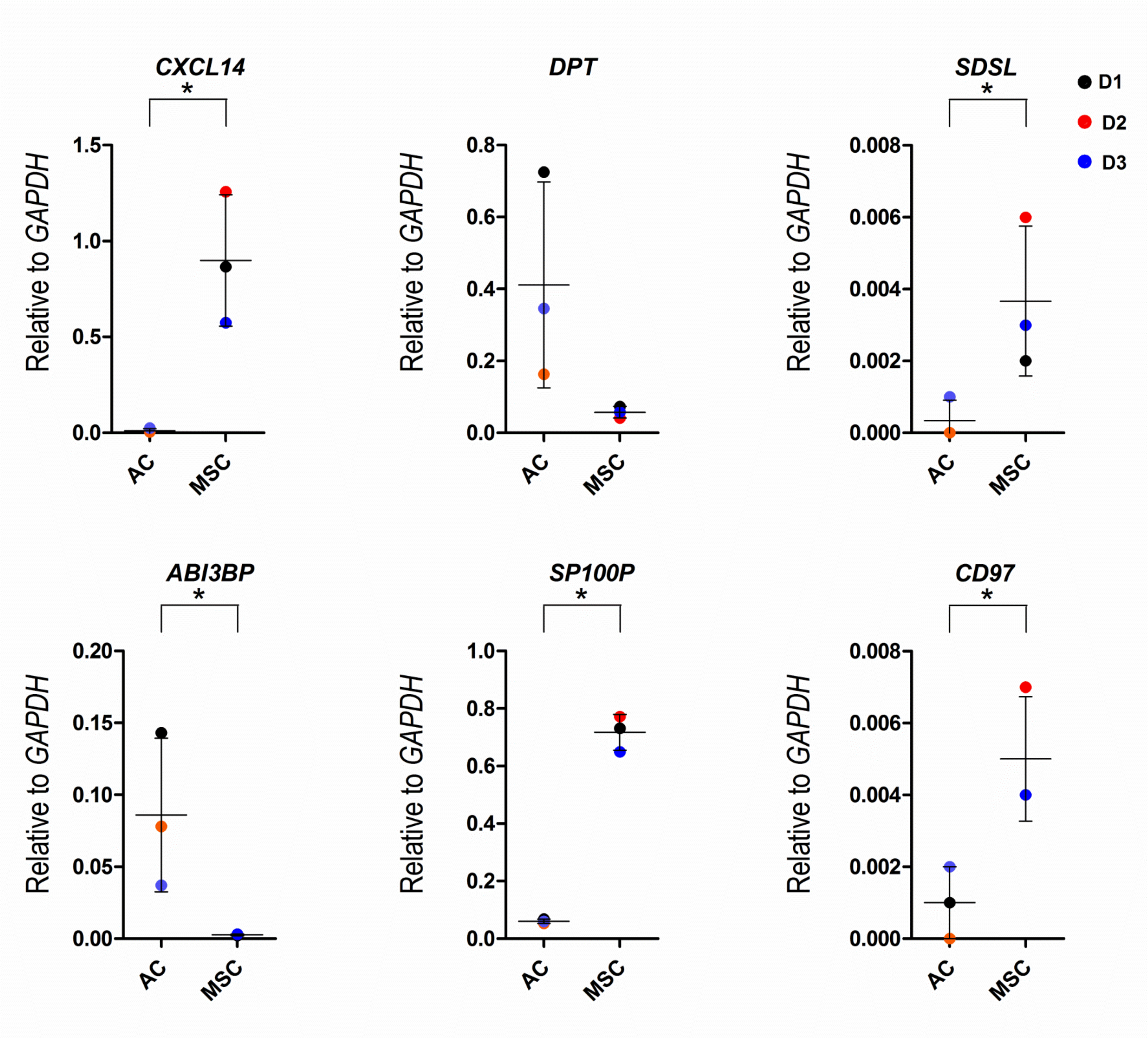

Supplementary Figure S7:
Gene expression in AC and MSC discs. Real-time RT-qPCR analysis of cartilage discs after 4 weeks of differentiation. Expression of selected genes to check candidates detected in the proteomics analysis. Data presents as dot plot diagram with mean and SD; D1-D3 (donor 1-3). Student's t-test was used for statistics, and $P \leq 0.05$ was considered significant. N = 3 for each experimental group and DF = 4.

Supplementary Figure S8

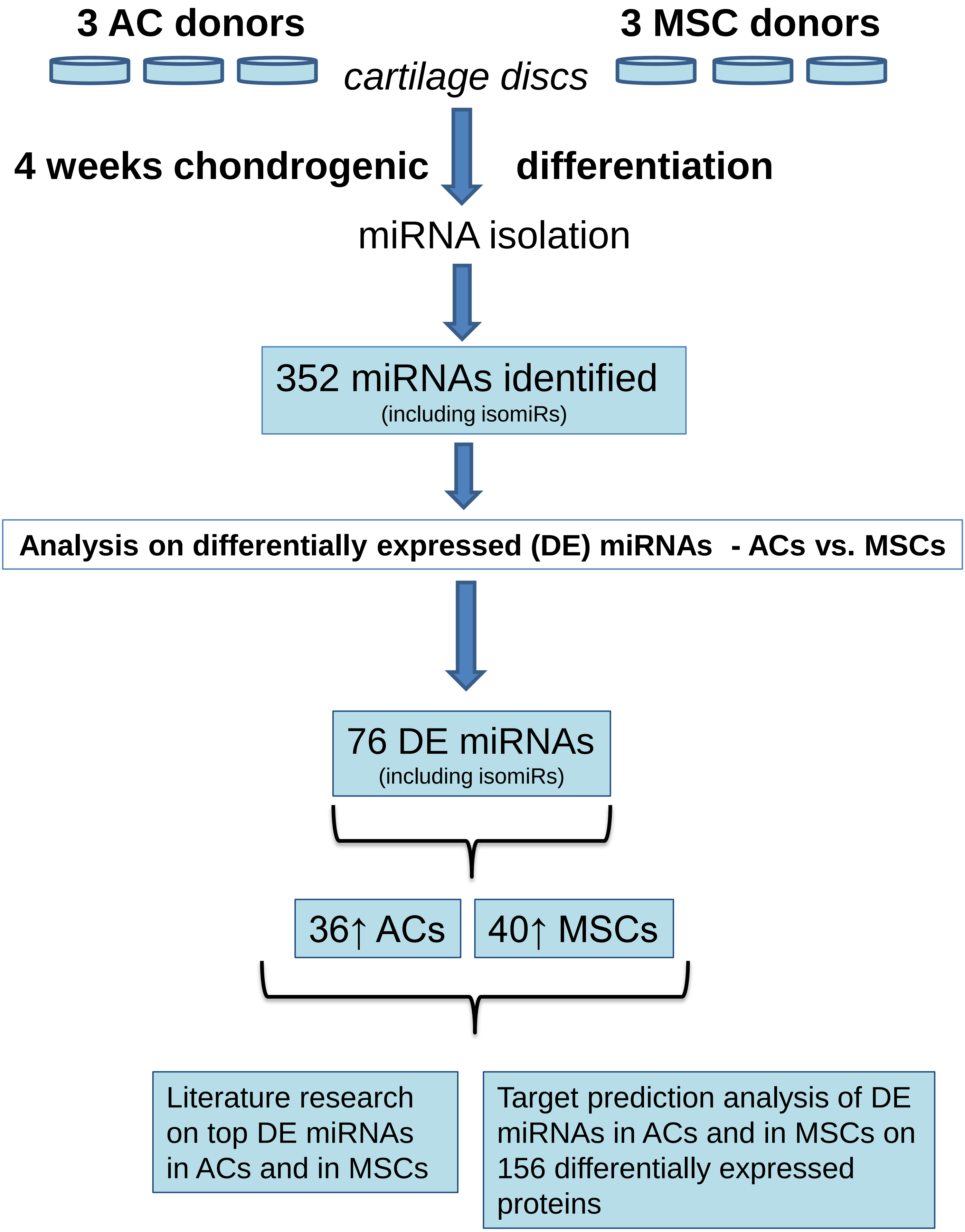

Supplementary Figure S8: Overview miRNA analysis.

Supplementary Materials and Methods

NGS data analysis

Raw data was analyzed using our FAIRPype analysis pipeline, downloadable as a jar file, together with configuration files, from https://github.com/CBGOUS/frekner_SmallRNAPipeline, together with all Python scripts listed below. The analysis is executed as a series of the following steps.

SingleReadAdapterTrim
The raw data was adapter trimmed with the step using the Trimmomatic software package (version 0.39) with the following parameters:

minAvgReadQual: 30,
minAlignScore: 7,
noOfMismatches: 2

and the adapter sequence 'AACTGTAGGCACCATCAAT', specified by Qiagen for their small RNA library kit.

ReadQualityFiltering
Sample quality of trimmed sequences was checked with the step using the FastQC, software package (version 0.11.9).

CollapseReads
Reads were converted to FASTA and collapsed using the fastq_to_fasta and the fastx_collapser tools within the FastX_Toolkit (version 0.0.13).

BowtieMapSingleReads
Read mapping was performed using the Bowtie software package (version 1.3.0). As the study was focusing on miRNAs and isomiRs, a reference index for read mapping was built based on the 1918 hairpin sequences present in v22.1 of miRBase. Overlapping entries (i.e., entries sharing identical or overlapping sequences with other entries) were removed using a Python script to produce a final set of 1743 unique hairpin sequences. A Bowtie index was generated using bowtie-index and mapping was performed with parameters -v 2

ParseSAMForMiRNAs
IsomiR read counting was performed with the 'bleed' parameter set to 2. i.e., any read that was within +/-2 nt of the specified start and stop position of the miRNA location specified in miRBase v21.1 was retained.

MergeIsomiRCounts
For read counting, isomiRs sharing the same seed region were counted together.

DEwithEdgeR
Estimation of differentially expressed features was performed using EdgeR using the classic Single Factor pipeline. The R code used to generate the analysis is also posted on GitHub

Target Prediction

Target Prediction was performed using the miRAW software package for a subset of predicted up and down regulated, with the miRAWwrapper Python script to generate the shell scripts for batch execution. The Pita model was selected and both canonical (seed region pairing) and non-canonical (extended seed region pairing) were retained
For 3'UTR targets, Ensembl reference annotation Homo_sapiens.GRCh38.102 was used. For genes with multiple annotated 3'UTRs, these were filtered to retain only (i) TSL1 or TSL2 support and (ii) the longest 3'UTR to give a final target set of 19362 3'UTRs.

Correlation of miRNA target predictions with protein expression data

As all miRNA target prediction tools have sub-optimal performance (i.e., predictions contain many False Positives and False Negatives) a Python script filterMiRAWpredictions.py was used to filter the prediction sets. Predicted targets with an estimated Mean Free Energy (MFE) between the miRNA and the mRNA < -15 or a target prediction probability < 0.99995 were removed.

Supplementary statistics information

	P-value	t-value
Disc thickness	0.01	5.56
Disc weight	0.00	6.68
Nuclei count	0.03	3.49
qPCR		
ACAN	0.63	0.52
Col1A1	0.00	6.31
Col10A1	0.05	2.77
Col2A1	0.92	0.10
CD44	0.01	4.78
COMP	0.07	2.41
PRG4	0.11	2.04
OGN	0.08	2.30
CILP2	0.04	3.02
ALPL	0.12	1.95
RUNX2	0.52	0.70
BGLAP	0.04	3.06
IBSP	0.02	3.53
SSP1	0.05	2.84
IHH	0.30	1.19
SP7	0.07	2.42
TMEM119	0.01	4.42
OMD	0.06	2.55
CXCL14	0.01	4.49
DPT	0.10	2.14
SDSL	0.03	3.17
ABI3BP	0.05	2.70
SP100P	0.00	18.21
CD97	0.02	3.94
miR-10a	0.02	3.87
miR-335	0.001	8.52
*miR-675**	0.04	3.04
miR-146a	0.02	4.00
miR-181a	0.04	3.04
miR-181b	0.001	8.52
